# Design and implementation of time-based fault tolerance technique for solar PV system reliability improvement in different applications

**DOI:** 10.1038/s41598-025-91464-4

**Published:** 2025-03-03

**Authors:** K. Karthik, P. Ponnambalam

**Affiliations:** School of Electrical Engineering, Vellore Institute of Technology, Vellore, Tamil Nadu India

**Keywords:** Photovoltaic (PV), Battery, Permanent magnet synchronous motor (PMSM), DC–DC converter, Inverter, Engineering, Energy science and technology

## Abstract

This paper investigates the application of time-based fault tolerance techniques in solar photovoltaic (PV), DC–DC converter, battery, and permanent magnet synchronous motor (PMSM) systems. The fault tolerance techniques are like open circuit switch-level, leg-level, module-level, and measurement-level. By leveraging time-based monitoring and analysis, these techniques enable early detection, isolation, and recovery from various faults, enhancing system reliability and availability. The study focuses on fault scenarios within the 0.15–0.3 time per/second frame, a critical window for rapid fault response. Specific techniques, including time-based fault detection, isolation, and recovery strategies are explored in detail. The OPAL-RT HIL testing platform is used to validate the simulation results and conduct tests to evaluate the efficiency of various methods. The results demonstrate the performance of the fault-tolerant systems and the implementation of effective time-based techniques in solar PV applications. Finally, this work can be useful for researchers who want to learn how solar PV systems, batteries, and PMSM systems behave in fault situations followed by the conclusion.

## Introduction

A crucial component of contemporary engineering systems is fault tolerance, especially in applications where availability and dependability are essential. It entails creating systems that can continue operating as intended, even with flaws or malfunctions. Engineers can greatly increase the resilience and robustness of systems through the use of several fault tolerance strategies. At the lowest level of the system, switch-level techniques concentrate on specific switches or gates. Gate-level methods consider how logic gates behave and how they are connected^1^. Using module-level approaches, entire modules or subsystems can be made redundant and fault-tolerant. Techniques at the measurement level include monitoring system parameters and using predictive and diagnostic maintenance techniques. Using these strategies, engineers can create systems that reduce downtime, manage errors gracefully, and guarantee continuous operation. Failures of switches, open circuits, and short circuits are examples of fault circumstances that might jeopardize PMSM systems^2^.

An important energy conversion component is a permanent magnet synchronous motor (PMSM), which offers numerous benefits like high torque density and efficiency. The drive system is asked to have fault tolerance in multiple industries. To attain fault tolerance (FT), the most widely utilized motor structures are multiphase and modular. Even if the multiphase motor is capable of FT operation, the methods and control system are complex. A single-phase fault results in a large torque ripple since we must adjust all the other healthy phase winding armature magnetomotive force (MMF). Multiple three-phase modules, each managed by a separate three-phase inverter, are part of the modular motor’s design. The health modules functioning normally won’t be impacted when a module fails; instead, it will be disconnected. In addition to using the established three-phase motor driving techniques, this Structure can individually control each module^3^. Thus, modular topology must be the main study area for fault-tolerant and highly reliable motors.

**Fig. 1:**
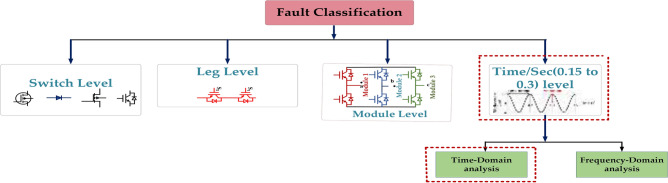
Fault classification of power electronics converter.

Figure 1 shows the many types of fault levels and four stages of fault classification. At the switch level, each transistor in a circuit is the main focus. Stuck-at faults, in which a transistor remains permanently at 0 or 1; bridge faults, in which two or more nodes are inadvertently connected; and open faults, in which a connection is broken, are examples of problems at this level^4^. Transistors that have short circuits between their source and drain terminals are examples of faults at this level. The open-circuit faults an internal connection is broken in the transistor. At the leg level, also known as the gate level, errors regarding logic gates are evaluated. The causes of faults may include bridging faults between gate inputs or outputs, stuck-at faults inside the gate, or incorrect gate output values^5^. A functional module, consisting of several gates or sub-modules, can have errors at the module level. Errors may manifest as stuck-at outputs, improper internal state transitions, or improper module behavior. One category of fault modeling and testing approaches used in digital circuit design is time-level fault procedures. They concentrate on defects that impact a circuit’s temporal behavior instead of its logical operation. Numerous variables, including aging, environmental influences, and manufacturing flaws, can cause these issues^6^. Classifying faults is not always simple, and there may be overlaps across many levels. For instance, a malfunction may appear as a module-level defect if the gate is essential to the module’s operation.

**Fig. 2:**
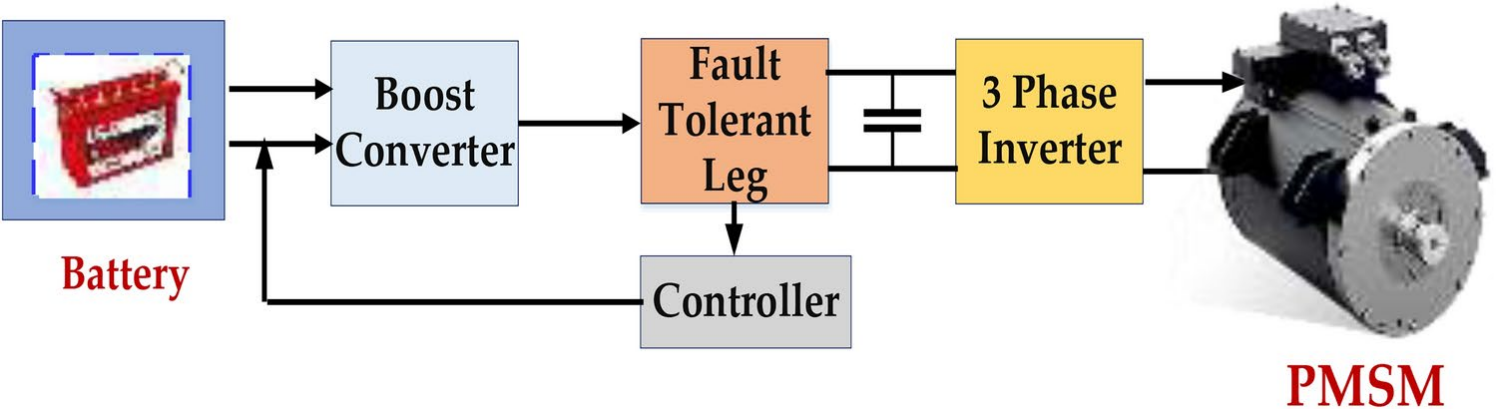
Block diagram for fault tolerance for PMSM with battery.

To ensure the dependable operation of battery energy storage systems (BESS) in Permanent Magnet Synchronous Motor drives, a fault-tolerant bidirectional converter, as shown in Fig. 2, is essential. This converter makes bidirectional power flow between the battery and the DC link possible, enabling both charging and discharging processes. Individual cell overcharging or over-discharging is avoided, and uniform aging is guaranteed via cell balancing^7^. Thermal management aims to prevent thermal Runaway by maintaining ideal operating temperatures. Fault detection and isolation: To identify and separate defective cells, cell voltages, temperatures, and currents must be monitored.

**Fig. 3:**
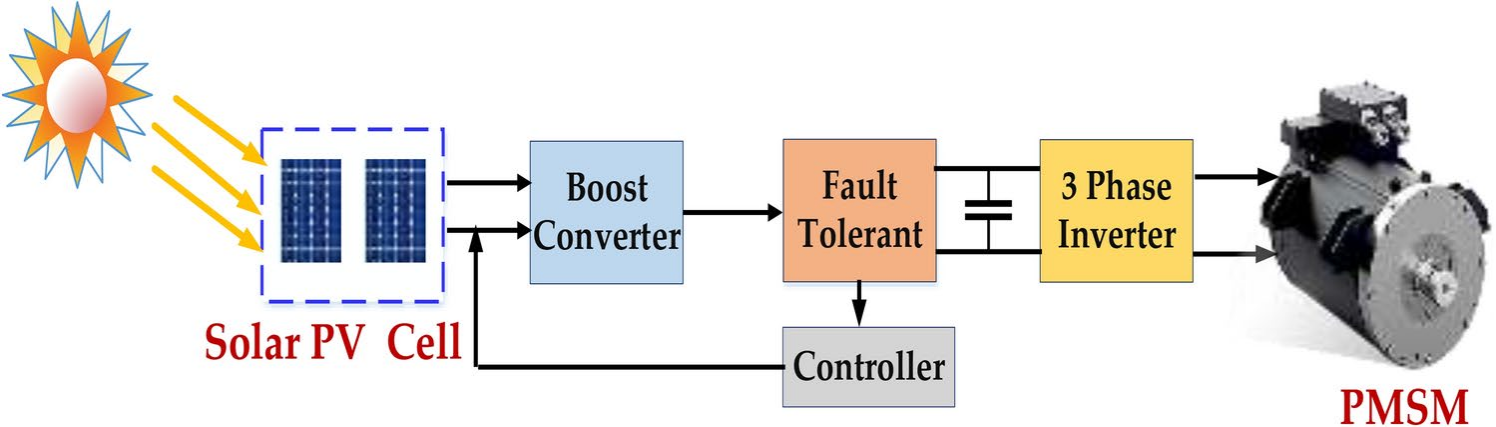
Block diagram for fault tolerance for PMSM with solar PV.

A typical solar PV system combined with a motor drive consists of a solar PV array, a DC–DC converter, and a motor drive, as shown in Fig. 3. The DC–DC converter enhances the DC power produced by the PV array to an appropriate voltage level system. The motor produces mechanical energy from electrical energy. Techniques for Monitoring Maximum Power Point (MPPT): The maximum power point of PV arrays that are partially shaded can be tracked using sophisticated MPPT algorithms^8^. The modular design divides the variety into smaller modules to provide targeted fault replacement and isolation.

The Table 1 represents the Summary of the Literature Review based on the parameters. The characteristics of various fault-tolerant leg-level, measure-level, and switch-level techniques are found in the literature. Each row represents a different technique. Overall, this table provides a quick overview of the key features and characteristics of different fault-tolerant leg-level techniques. It can compare and contrast these techniques based on various criteria such as power density, complexity, efficiency, and fault tolerance capabilities.

**Table 1:** Summary of literature review.

References	Objective	Methodology	Technique	Performance	Results and observation	Limitations
^9^	Improve efficiency and reduce switching losses	Simulation and experimental analysis	Finite control set model predictive control	Total harmonic distortion (THD), switching losses	Simulation and experimental results demonstrate improved efficiency and reduced switching losses compared to conventional methods	Computational burden, complexity of implementation
^10^	Improve transient response and reduce overshoot/undershoot	Simulation and experimental analysis	Proportional-integral (PI) controller with feedforward compensation	Measured transient response compared to theoretical values	Sensitivity to parameter variations, tuning challenges	Higher cost, increased complexity
^11^	Improve steady-state performance	Simulation and experimental analysis	PI controller	Steady-state error, response time	Simulation and experimental results compared to theoretical values	Higher computational complexity, sensitivity to parameter variations
^12^	Reduce harmonic distortion and improve efficiency	Simulation and experimental analysis	Modulation techniques (e.g., space vector modulation	THD analysis, efficiency measurements	Simulation and experimental results demonstrate reduced THD and improved efficiency	Increased complexity, higher component count
^13^	Improve efficiency and reduce switching losses	Simulation and experimental analysis	Matrix converter control algorithms	Switching losses, efficiency, and stability analysis	The complexity of control and implementation, the potential for instability	Slower response, potential for overshoot
^14^	Improve steady-state performance and dynamic response	Simulation and experimental analysis	Vector control techniques	Steady-state performance, dynamic response, robustness to parameter variations	Sensitivity to parameter variations, the potential for instability	Lower cost, increased complexity
^15^	Improve efficiency and reduce switching losses	Simulation and experimental analysis	PI controller design and tuning	Efficiency, switching losses, response time	Sensitivity to parameter variations, limited bandwidth	Higher computational burden, sensitivity to parameter variations
^16^	Improve robustness to parameter variations and disturbances	Simulation and experimental analysis	Fuzzy logic control algorithms	Robustness to disturbances, fault tolerance, tuning complexity	Tuning complexity, potential for instability	Computational burden, complexity of implementation
^17^	Improve stability and transient response	Simulation and experimental analysis	Lead-lag compensator design and tuning	Bandwidth limitations, potential for oscillations	Simulation and experimental results compared to theoretical values	Slower response, potential for overshoot
^18^	To develop efficient replication strategies for distributed systems	Analytical approach and simulations	Replication algorithms and optimization methods	Throughput, latency, overhead	Reduced latency by 30% compared to traditional methods	Struggles with network partition scenarios, potentially leading to data inconsistencies
^19^	Fault Detection & Isolation (FDI) in rotating machinery	Time-domain analysis	Vibration signal analysis, Statistical methods	Vibration amplitude, frequency content, statistical parameters	Effective in detecting bearing faults, imbalance, misalignment	Computational burden, complexity of implementation
^20^	To apply machine learning for load balancing	Machine learning models and simulations	ML-based load-balancing techniques	ML-based load-balancing techniques	Enhanced load distribution efficiency by 25% compared to static methods	High computational cost for model training and inference
^21^	Fault diagnosis in power systems	Frequency-domain analysis	Fourier transform, spectral analysis	Frequency spectrum, harmonic content, power spectral density	Detection of power quality issues, harmonics, and system instabilities	Potential for overmodulation, sensitivity to parameter variations
^22^	Fault detection in aerospace systems	Time–frequency analysis	Wavelet transform, short-time Fourier transform	Time–frequency representation of signals	Detection of transient faults, analysis of non-stationary signals	Potential for overmodulation, sensitivity to parameter variations
^23^	Structural health monitoring	Time-domain analysis	Wavelet transform, short-time Fourier transform	Changes in natural frequencies, damping ratios	Detection of cracks, damage, and degradation in structures	Tuning complexity, potential for instability
^24^	To provide scalable fault tolerance through checkpointing and load balancing	Proposes a combined approach using checkpointing and load balancing to achieve scalable fault tolerance	Integrates checkpointing mechanisms with load balancing to provide robust fault tolerance	Fault tolerance, load balancing efficiency, checkpointing overhead	Reduced recovery time by 20% and increased system throughput by 25%	Overhead from frequent checkpointing and dynamic balancing

The Table 2 represents the provides a quick overview of the key features and characteristics of different fault-tolerant leg-level techniques. It can be used to compare and contrast these techniques based on various criteria such as power density, complexity, efficiency, and fault tolerance capabilities. The specific to each technique and may vary depending on the operating conditions and design parameters. The Power density indicates the power output per unit weight of the converter. Complexity describes the complexity level of the control system and its implementation. The Control methodology specifies the control method used (e.g., Predictive torque control, PI control).

**Table 2:** Comparison of the different fault-tolerant switch-level techniques.

References	Input voltage	Merits	Demerits	Output voltage	Efficiency (%)	Power density	Control complexity	Topology	Control method
^11^	200–280 V_AC_ (3*φ*)	High power density, fast response	High power density, fast response	101 V_DC_	85–90	300	High	3*φ* AC–DC	Speed/current control
^12^	220 V_AC_ (3*φ*)	Good transient response	Potential for overshoot and undershoot	400 V_DC_	80–85	150	High	3*φ* AC–DC	Current control
^13^	220 V_AC_ (3*φ*)	Simple control implementation	Slower response compared to the current control	400 V_DC_	80–85	180	High	3*φ* AC–DC	Voltage control
^14^	100 V_AC_ (3*φ*)	Reduced harmonic distortion, improved efficiency	Higher complexity compared to conventional rectifiers	200 V_DC_	85	150	Medium	Vienna rectifier	PI controller
^25^	50 V_AC_ (3*φ*)	Reduced switching losses, lower voltage stress on devices	Higher complexity, potential for instability	60 V_AC_ (3*φ*)	82	20	Medium	Reverse matrix	PI controller
^15^	200–250 V_AC_ (3*φ*)	Good performance in steady-state	an be sensitive to parameter variations	600 V_DC_	80–85	150	Medium	3*φ* AC–DC	Voltage oriented control
^17^	300 V_DC_	Simple and robust	Slower response, potential for overshoot	380 V_AC_ (3*φ*)	86	25	Low	3*φ* inverter	PI controller
^20^	11 kV AC (3*φ*)	Robustness to parameter variations, adaptability	Tuning complexity, potential for instability	20 kV_DC_	90–95	250	Low	3*φ* AC–DC	Fuzzy

Table 3 represents the table and provides a comparative analysis of different fault-tolerant leg-level techniques, allowing for a quick assessment of their strengths and weaknesses based on various parameters. The input Voltage specifies the voltage level at which the converter receives power. The output voltage specifies the voltage level at which the converter provides power, and efficiency represents the ratio of output power to input power, indicating the converter’s energy conversion efficiency. The Power measures the power output per unit weight of the converter, indicating its compactness and efficiency. The Control method specifies the control algorithm used to regulate the power flow and maintain stability. It describes the level of complexity involved in implementing and tuning the control algorithm.

**Table 3:** Comparison of the different fault-tolerant leg-level techniques.

References	Input voltage	Merits	Demerits	Output voltage	Efficiency (%)	Power density	Control complexity	Topology	Control method
^26^	200 V_AC_ (3*φ*)	High power density, good performance	Complex topology, higher component count	400 V_DC_	85	500	High	6-Leg AC–DC–AC	Voltage oriented control
^27^	60 V_AC_ (3*φ*)	High power density, reduced switching losses	Complex topology, higher component count	50 V_AC_ (3*φ*)	80–90	120	High	5-Leg Converter	Voltage oriented control
^28^	230–440 VAC (3*φ*)	Robustness to parameter variations, good performance	Higher complexity, tuning challenges	600 V_DC_	85–90	222	Low	3*φ* AC–DC	PI Controller
^29^	190 V_AC_ (3*φ*)	High efficiency, fast dynamics, reduced common-mode voltage	Increased complexity, the potential for instability	400 V_DC_	80–85	80	Medium	Bidirectional AC/DC	Model Predictive Direct Power Control
^30^	190 V_AC_ (3*φ*)	High efficiency, fast dynamics, reduced common-mode voltage	Increased complexity, potential for instability	400 V_DC_	85	100	Medium	Bidirectional AC/DC	Model Predictive Direct Power Control
^28^	50 V_AC_ (3*φ*)	High efficiency, fast dynamics, reduced common-mode voltage	Increased complexity, the potential for instability	300 V_DC_	85	50	High	Bidirectional AC/DC	Finite State Model

The different fault-tolerance measurement-level technique is shown below in Table 4. The input Voltage is Specifies the voltage level at which the converter receives power. The Output Voltage: Specifies the voltage level at which the converter provides power. Efficiency represents the ratio of output power to input power, indicating the converter’s energy conversion efficiency. The Power density measures the power output per unit weight of the converter, indicating its compactness and efficiency. The control method is Specifies the type of control algorithm used to regulate the power flow and maintain stability. The Control Complex describes the complexity level of implementing and tuning the control algorithm.

**Table 4:** Comparison of the different fault-tolerant measure-level techniques.

References	Input voltage	Merits	Demerits	Output voltage	Efficiency (%)	Power density	Control complexity	Topology	Control method
^31^	200 V_AC_ (3*φ*)	High efficiency, modularity	Voltage balancing issues	400 V_DC_	80–85	100	Low	3*φ* AC–DC	PI controller
^32^	220 V_AC_ (3*φ*)	Reduced switching losses, improved efficiency	Voltage balancing issues	360 V_DC_	80–90	110	Low	3*φ* AC–DC–AC	PI controller
^32^	110 kV_AC_	High voltage capability, reduced stress on devices	High cost, complex control	400 kV_DC_	88	800	Medium	Multi-terminal high-voltage	Multi-terminal high-voltage direct current
^31^	600 V_AC_ (3*φ*)	Improved robustness, reduced sensitivity to disturbances	Higher complexity, tuning challenges	380 V_DC_	80–90	150	Low	Hybrid AC–DC	Sliding modes controller
^33^	220 V_AC_ (3*φ*)	Simple and robust, low cost	Slower response, potential for overshoot	400 V_DC_	70–80	180	Low	1-Phase PWM rectifier	PI control
^34^	100 V_DC_	Improved stability, reduced overshoot	Limited bandwidth, potential for oscillations	220 V_DC_	80–90	140	Low	PWM inverter	Adaptive control

The remainder of this paper is organized as follows: Section 2 describes the mathematical model of the modular motor with n modules. Section 3 investigates fault-tolerance inverter Topologies for leg-level techniques. Then, Section 4 discusses lithium-ion battery fault conditions. Section 5 Shows open circuit-switch fault tolerance in the inverter. Section 6 shows the Simulation and OPAL-RT results, and Section 7 shows the experimental setup. Section 8 presents a brief conclusion.

## Mathematical model for modular motor with N modules

The motor’s stator is segmented into modules around its circumference. A collection of separately operated three-phase windings makes up each stator module. The stator windings of every module in the motor system are all three-phase Y-connected symmetrical windings, assuming there are n modules. The electrical position of each module is identical, as is the electrical angle of the corresponding phase^35^. This motor feature is demonstrated. Therefore, the three-phase current equation is the same for each module.

According to the analysis, each model motor’s FOC strategy functions normally. The motor control topology uses a sensible torque distribution control technique to reduce the motor’s torque control to the current power of each module.

1 $$\begin{aligned} i_{A1,2 \ldots n} & = I_{m1,2 \ldots n} \left( {\cos ( \cdot \omega_{e} t} \right) \\ i_{B1,2 \ldots n} & = I_{m1,2 \ldots n} \left( {\cos ( \cdot \omega_{e} t) - \frac{2}{3}\pi } \right) \\ i_{C1,2 \ldots n} & = I_{m1,2 \ldots n} \left( {\cos ( \cdot \omega_{e} t) + \frac{2}{3}\pi } \right) \\ \end{aligned}$$

where I_m1,2,3…n_ is the amplitude of the phase current of each module, and e is the electrical angular frequency.

In the d–q coordinate system, the voltage equation of the modular motor is

2 $$\left( {\begin{array}{*{20}c} {U_{dq1} } \\ \cdot \\ {U_{dqn} } \\ \end{array} } \right) = \left[ {\begin{array}{*{20}c} {A_{1} } & \cdots & 0 \\ \vdots & \ddots & \vdots \\ 0 & \cdots & {A_{n} } \\ \end{array} } \right]\left( {\begin{array}{*{20}c} {I_{dq1} } \\ \cdot \\ {I_{dqn} } \\ \end{array} } \right) + \left[ {\begin{array}{*{20}c} {B_{1} } & \cdots & 0 \\ \vdots & \ddots & \vdots \\ 0 & \cdots & {B_{n} } \\ \end{array} } \right]X\left( {\begin{array}{*{20}c} {I_{dq1} } \\ \cdot \\ {I_{dqn} } \\ \end{array} } \right) + \left( {\begin{array}{*{20}c} {C_{1} } \\ \cdot \\ {C_{n} } \\ \end{array} } \right)$$

where *U*_dq1,2…n_ = [*U*_d1,2…n_ *U*_q1,2…n_]^T^*U*_q1;2…n_, *U*_d1,2…n_ are the *q*- and *d*-axis voltages of each module, respectively *I*_dq1,2…n_ = [*I*_d1,2…n_ *I*_q1,2…n_]^*T*^, *I*_q1;2…n_, *I*_d1;2…n_ are the *q*- and *d*-axis currents of each module, respectively.

$$\begin{aligned} {\mathbf{A}}_{{1,2 \ldots {\text{n}}}} &= \left[ {\begin{array}{*{20}c} {R{\text{s}}1,2 \ldots {\text{n}}} & { - L{\text{q}}1,2 \ldots {\text{n}}\omega {\text{e}}} \\ {L{\text{d}}1,2 \ldots {\text{n}}!{\text{e}}} & {R{\text{s}}1,2 \ldots {\text{n}}} \\ \end{array} } \right], \hfill \\ {\mathbf{B}}_{{1,2 \ldots {\text{n}}}} &= \left[ {\begin{array}{*{20}c} {L{\text{d}}1,2 \ldots {\text{n}}} & 0 \\ 0 & {L{\text{q}}1,2 \ldots {\text{n}}} \\ \end{array} } \right], \hfill \\ {\mathbf{C}}_{{1,2 \ldots {\text{n}}}} &= \left[ {\begin{array}{*{20}c} \cdot & 0 \\ \cdot & {\omega {\text{e}}\psi {\text{f}}} \\ \end{array} } \right], \hfill \\ \end{aligned}$$

*R*s1, 2…n, *L*d1,2…n and *L*q1,2…n are the phase winding resistance, *q*- and *d*-axis the inductance of each module, and f is the PM flux linkage.

3 $$Te=\sum_{i=1}^{n}\left({Te}_{i}=\frac{3}{2}\text{p}\left\{\mathit{\psi f}\sum_{i=1}^{n}{\mathit{Iq}}_{i}+\sum_{i=1}^{n}\left[\left(\mathit{Ldi}-\mathit{Lqi}\right)\right]\mathit{IdiIqi}\right\}.\right)$$

where *T*e is the modular module’s output torque and indicates.

**Fig. 4:**
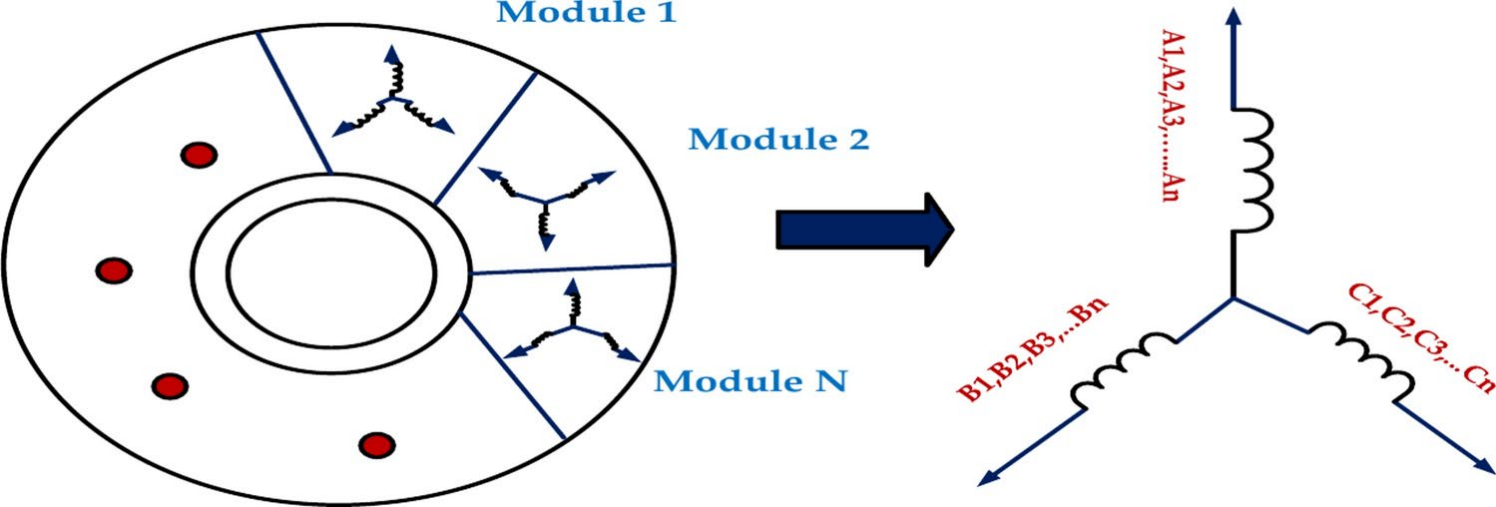
Equivalent circuit for modular motor with N module.

The study mentioned above makes it abundantly evident that we can regulate the modular motor while it operates normally by using the field-oriented control method for each module^36^. This is the Modular Motor Control System Topology. This makes it possible to simplify the torque management of the modular motor to the current control of each module using a sensible torque distribution control approach. Figure 4 represents the modular motor with N modules to determine the voltage, current, and equal inductor. This is a simplified equivalent circuit. The motor’s behavior may be more complex due to magnetic coupling between modules, back EMF, and non-linear effects.

**Fig. 5:**
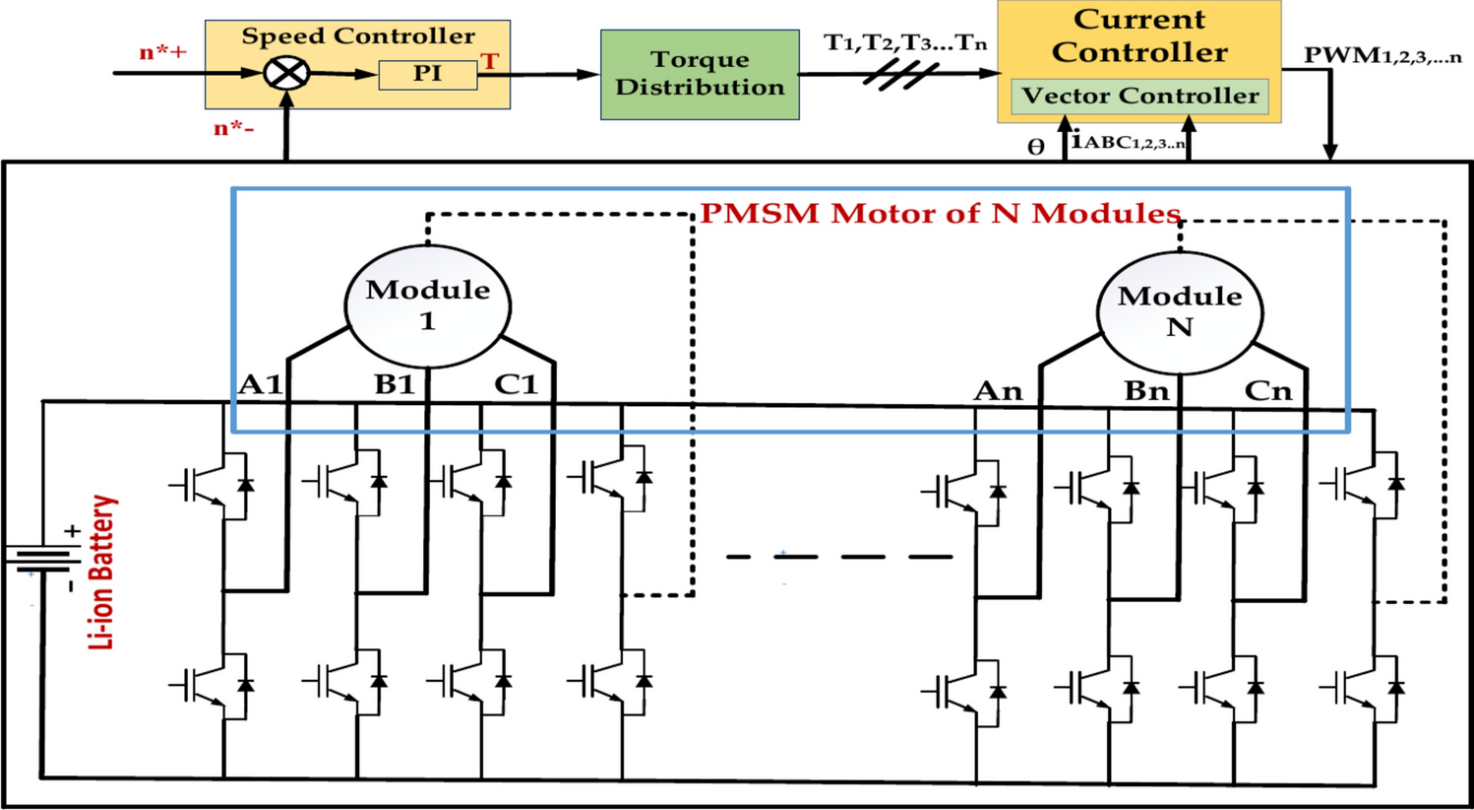
Modular motor control system.

The module motor consists of eight modular pieces with three phases. It consists of two sets of three-phase Y-connected windings per module. Phase windings that are open circuits are disconnected from the system. The other set of windings functions similarly to conventional three-phase windings^37^. A very minimal magnetic connection exists between units. When the motor malfunctions, the faulty module may surely be removed from the system, cutting off the defective module can be realized, and the system can achieve fault-tolerant operation, but it significantly reduces its output capability. For example, if there are two modules, the output capability will be reduced by 1/2 per Unit; if there are n modules, the output capability will be decreased by 1/n per Unit^38^. Figure 5 represents the Modular motor control system for a permanent magnet synchronous motor with N modules. The module controls the speed, Torque, and current vector controller. The PI controller is commonly used for speed control. It calculates the error between the desired and actual speeds and generates a control signal to drive the motor to the desired speed.

Table 5 represents the normal operation conditions here. The observations module contributes approximately half of the total motor torque. The current levels of both modules are similar, indicating balanced operation. The motor is operating below its rated torque, suggesting headroom for increasing performance. By analyzing these parameters and potentially conducting further investigations, engineers can optimize the motor design and control strategies to improve its overall performance and efficiency.

**Table 5:** Under normal operation and motor parameters^39^.

Under normal operation				Motor parameters	
Module number	var	Value	p.u Value	Rated torque	3 N m
Module 1	Iq	3.54 A	1		
	Im	3.69 A	1	Number pole Pairs	4
	Te	1.53 N m	1	Phase winding resistance	0.5 Ω
Module 2	Iq	3.58 A	1	d-axis inductance	23.8 mH
	Im	3.67 N m	1	q-axis inductance	42.8 mH
	Te	1.55 N m	1		
Total motor	Te	3.04 N m	1	PM flux inductance	0.072 Wb

### Open-circuit fault-tolerance strategy for motor using module-level techniques

#### Single-phase open circuit fault-tolerant strategy for modular motor

A single-phase open-circuit failure will affect the inverter’s bridge arm or motor’s single phase. Both kinds of open-circuit faults can be handled using the same fault-tolerant approach. The MMF compensation approach is therefore used to address the single-phase open-circuit defect in a single module, in accordance with the modular motor’s property, which is that each module can be considered a comparable three-phase motor^40^. When the fault happens, the amplitude of the basic magnetic flux produced by the associated module in the air gap reduces and becomes unconstant, which causes a big torque ripple and a decrease in torque. Using phase A1 in Module 1 open as an example, phase B1 and C1 currents can be changed in accordance with the MMF principle to offset the MMF drop brought on by an open-circuit fault, thereby lessening the negative impact of phase A1.

Under normal conditions, the function of a three-phase symmetrical winding in a single module is:

4 $$\begin{aligned} F_{n1} & = f_{A1} + f_{B1} + f_{C1} \\ \text{F}_{\text{n}1} & = \frac{3}{2}{\text{NIm}} 1 \, \cos \left( {\upomega \text{et}} \right) \, \cos \left( {\uptheta\text{s}} \right) + \frac{3}{2} {\text{NIm}} 1 \, \cos \left( {\upomega\text{et}} \right) \, \cos \left( {\uptheta\text{s}} \right) \\ \end{aligned}$$

where *N* is the number of turns per phase in series θs is the electrical angle between the current position and winding A1 axis.

5 $$\text{F}_{1\text{ph-oc}} = \text{NiB}_{1} \cos \left( {\uptheta\text{s} - \frac{2\uppi }{3}} \right) + \text{Nic}_{1} \cos \left( {\uptheta\text{s} - \frac{4\uppi }{3}} \right)$$

6 $$\text{F}_{1\text{ph-oc}} = \left( { - \frac{1}{2}\text{iB}_{1} - \frac{1}{2}\text{ic}_{1} } \right)\text{N}\cos \uptheta\text{s} + \left( {\frac{\sqrt 3 }{2}\text{iB}_{1} - \frac{\sqrt 3 }{2}\text{ic}_{1} } \right)\text{N}\sin \uptheta\text{s}$$

where *i*B1 and *i*C1 are the currents of phase B1 and C1 after the fault occurred, respectively. Assuming that the increase of current will not cause the saturation in the cores, then letting (4) equal to (6), we get

7 $$\begin{aligned} \text{iB}_{1} = \surd{3}{\text{Im}} \cos \left( {\upomega\text{et} - \frac{5\uppi }{6}} \right) \hfill \\ \text{ic}_{1} = \surd{3}{\text{Im}} \cos \left( {\upomega\text{et} - \frac{5\uppi }{6}} \right) \hfill \\ \end{aligned}$$

The zero-sequence current can be given by

8 $$\text{io} = \frac{1}{3}(\text{iA}_{1} + \text{iB}_{1} + \text{ic}_{1} ) = - \surd{3}{\text{Im}} 1 \, \cos (\upomega\text{et})$$

**Fig. 6:**
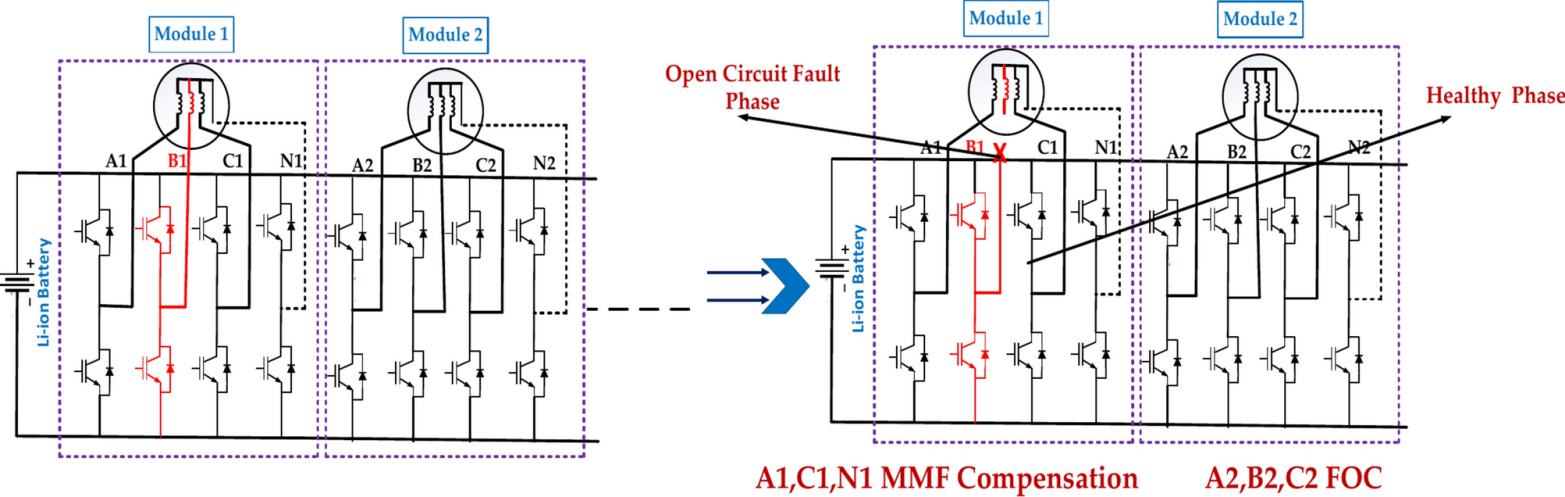
The diagram represents the faulty module under the single-phase open circuit fault.

Figure 6 illustrates the “MMF Compensation” concept under the single-phase open circuit fault. It depicts a power system with multiple transformers (represented by vertical lines with horizontal lines) connected in parallel. Each transformer has its magnetizing current (MMF) flowing through it. The MMF side shows the same power system, but now it has additional components (the diagonal lines) connected across the transformers^41^. These components are designed to compensate for the differences in magnetizing currents between the transformers. This technique ensures that all transformers in a parallel system share the load equally. Without compensation, transformers with lower MMF would carry a disproportionate amount of the load, potentially leading to overheating and damage. What are the observable effects of the fault, such as voltage drops, power outages, or unusual noises.

The single-phase Open circuit fault traditional and extraction for fault tolerance Table 6 represents the data for two different fault tolerance circuits, i.e., TOCFTC and EOCFTC, during a single-phase open circuit fault. The EOCFTC demonstrates superior fault tolerance to the traditional TOCFTC by allowing limited operation from the faulted module. The EOCFTC maintains a higher level of torque output during the fault condition. The EOCFTC introduces some level of complexity in control and may result in slightly higher errors in parameter values.

**Table 6:** Single-phase open circuit fault traditional and extraction for fault tolerance^42^.

Single-phase open circuit Fault								
Traditional open-circuit fault tolerance circuit (TOCFTC)					External open-circuit fault tolerance circuit (EOCFTC)			
Module number	Var	Value	Actual p.u value	Theoretical p.u value	Value	Actual p.u value	Theoretical p.u value	Error (%)
Module 1	Iq	0	0	0	2.08 A	0.587	0.577	1.70
	Im	0	0	0	4.02 A	1.1	1	9.00
	Te	0	0	0	0.91 N m	0.588	0.577	1.80
Module 2	Iq	3.74 N m	1.04	1	3.68 A	1.03	1	2.90
	Im	3.98 N m	1.08	1	3.76 A	1.05	1	4.70
	Te	1.61 N m	1.04	1	1.58 N m	1.03	1	2.90
Total motor	Te	1.61 N m	0.52	0.5	2.49 N m	0.809	0.788	2.50

#### Two-phase open circuit fault of the PMSM motor

**Fig. 7:**
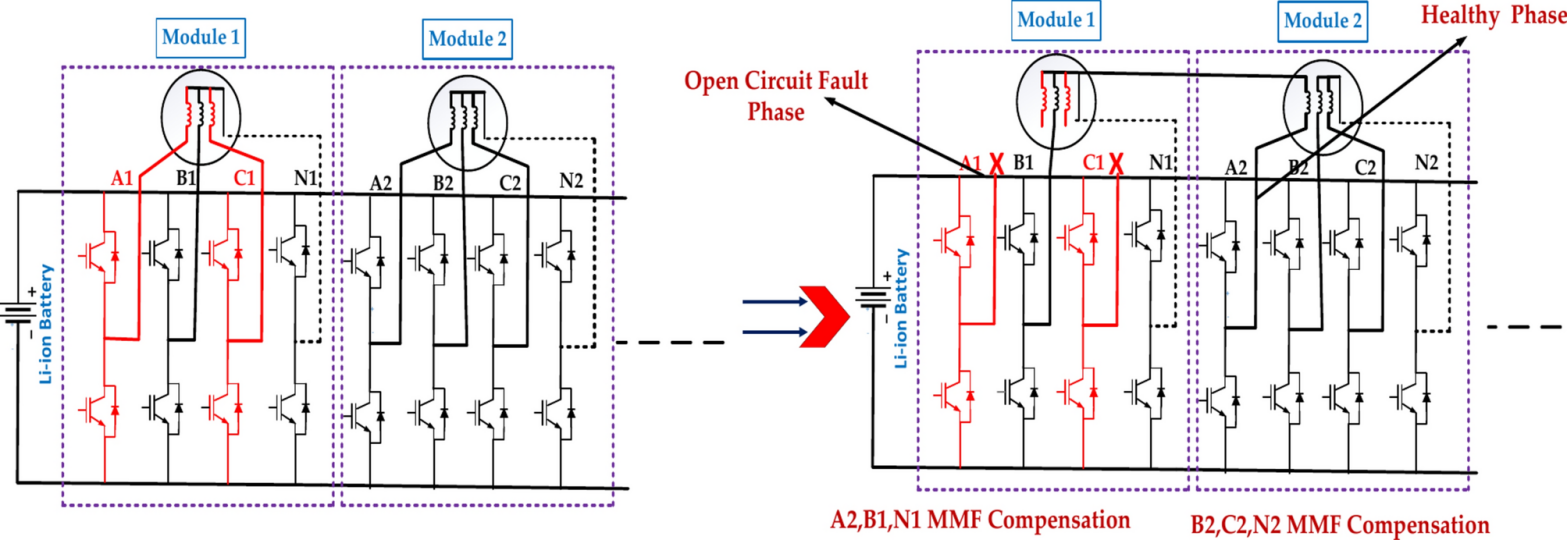
The structure of the faulty module under the two-phase open-circuit fault in the first case.

Figure 7 represents a two-phase open-circuit fault in a power system. This is a severe condition in which two phases lose electrical connection, typically due to a broken conductor or faulty switch. This can have significant implications for the system’s operation and stability. The specific Structure of the defective module will depend on the type of power system used^43^. The exact location of the fault within the module will determine the extent of the damage and the required repair actions. The effectiveness of the protection system in detecting and isolating the fault will impact the overall system reliability. The above diagram represents the A2, B1, and N1 MMF Compensation to B2, C2, and N2 MMF compensation for the open circuit fault and healthy phases.

**Fig. 8:**
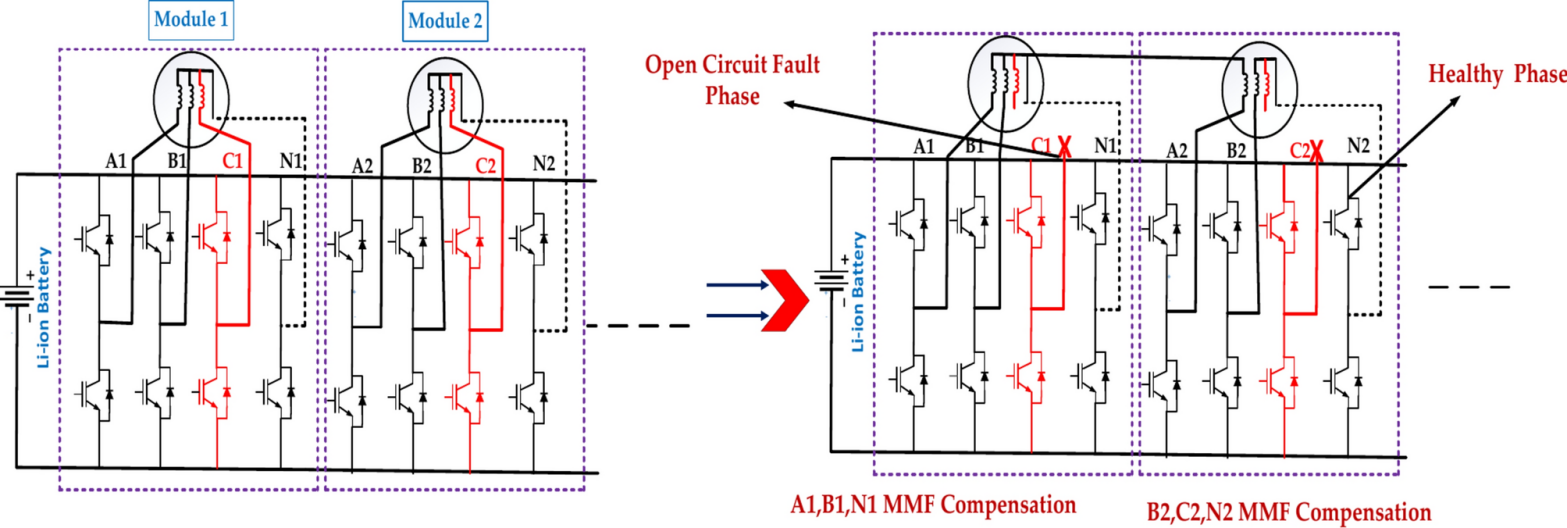
The structure of the faulty module under the two-phase open-circuit fault is in the second case.

Figure 8 represents the fault module under the two open circuit faults in the second case. The exact location of the fault within the module will determine the extent of the damage and the required repair actions. The effectiveness of the protection system in detecting and isolating the fault will impact the overall system reliability. The above diagram represents the A1, B1, and N1 MMF Compensation to B2, C2, and N2 MMF compensation for the open circuit fault and healthy phases.

Table 7 represents the Two-phase circuit fault for EOCFTC. This analysis is solely based on the provided data. A more thorough evaluation would require detailed data. This analysis is for informational purposes only and should not be considered. There are several impacts of a two-phase open circuit fault on a motor. While EOCFTC provides some level of fault tolerance, it is crucial to implement robust fault detection and isolation mechanisms to minimize the impact of such faults.

**Table 7:** Two-phase circuit fault for EOCFTC.

Two-phase open circuit fault					
(External open-circuit fault tolerance circuit (EOCFTC)					
Module number	Var	Value	Actual p.u value	Theoretical p.u value	Error (%)
Rec. Module 1	Iq	2.13 A	0.6	0.577	1.70
	Im	4.11 A	1.119	1	9.00
	Te	0.92 N m	0.601	0.577	1.80
Rem. Module 2	Iq	2.17 A	0.606	0.577	2.90
	Im	4.18 A	1.132	1	4.70
	Te	0.93 N m	0.606	0.577	2.90
Total motor	Te	1.85 N m	0.601	0.577	2.50

#### Three-phase open circuit fault of the PMSM motor

**Fig. 9:**
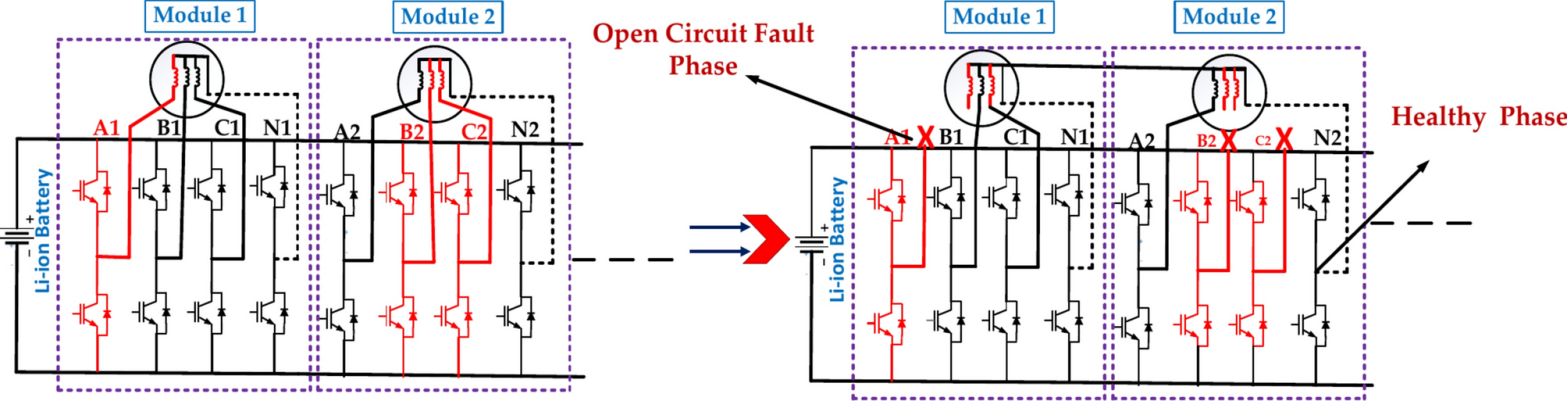
The structure of the faulty module under the three-phase open-circuit fault is in the second case.

Figure 9 represents the fault module under the open circuit faults in the second case. The exact location of the fault within the module will determine the extent of the damage and the required repair actions. The effectiveness of the protection system in detecting and isolating the fault will impact the overall system reliability. There is the open circuit fault phase and the healthy phase.

**Fig. 10:**
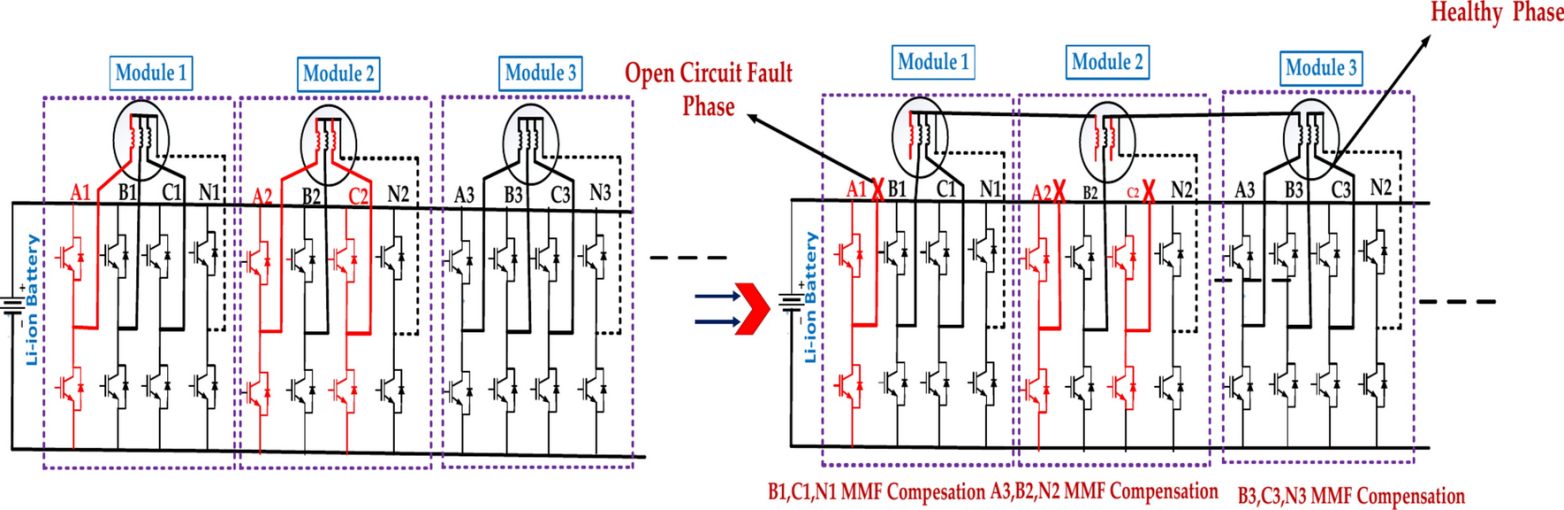
The structure of the faulty module under the three-phase open-circuit fault is in the third case.

Figure 10 represents the fault module under the three open circuit faults in the third case. The exact location of the fault within the module will determine the extent of the damage and the required repair actions^44^. The effectiveness of the protection system in detecting and isolating the fault will impact the overall system reliability. The above diagram represents the B1, C1, and N1 MMF Compensation, A3, B2, N2, MMF Compensation, B3, C3, and N3 MMF Compensation for the open circuit fault and healthy phases^45^.

Table 8 represents the Three-phase open circuit faults with an EOCFTC. Three-phase and multiphase open circuit faults pose significant challenges to motor operation. The EOCFTC can provide some level of fault tolerance, but the motor’s performance will be significantly degraded. Robust fault detection and isolation mechanisms are crucial to minimize the impact of these faults. The "Multiphase Open Circuit Fault" is limited. It would be beneficial to have more detailed information to understand better the motor’s behavior under such severe fault conditions.

**Table 8:** Three-phase and multiphase open circuit faults.

Module number	Three-phase open circuit Fault				Multiphase open circuit Fault			
	(External open-circuit fault tolerance) circuit (EOCFTC)				(External open-circuit fault tolerance) circuit (EOCFTC)			
	Var	Value	Actual p.u value	Theoretical p.u value	Value	Actual p.u value	Theoretical p.u value	Error (%)
Rec. Module 1	Iq	3.71 A	1.04	0.577	2.32 A	0.655	0.577	13.50
	Im	3.81 A	1.03	1	4.41 A	1.195	1	19.50
	Te	1.60 N m	1.04	0.577	1.00 N m	0.655	0.577	13.50
Total motor		1.60 N m	0.52	0.577	1.00 N m	0.325	0.289	12.40

#### Extreme multiphase open circuit fault of the PMSM motor

**Fig. 11:**
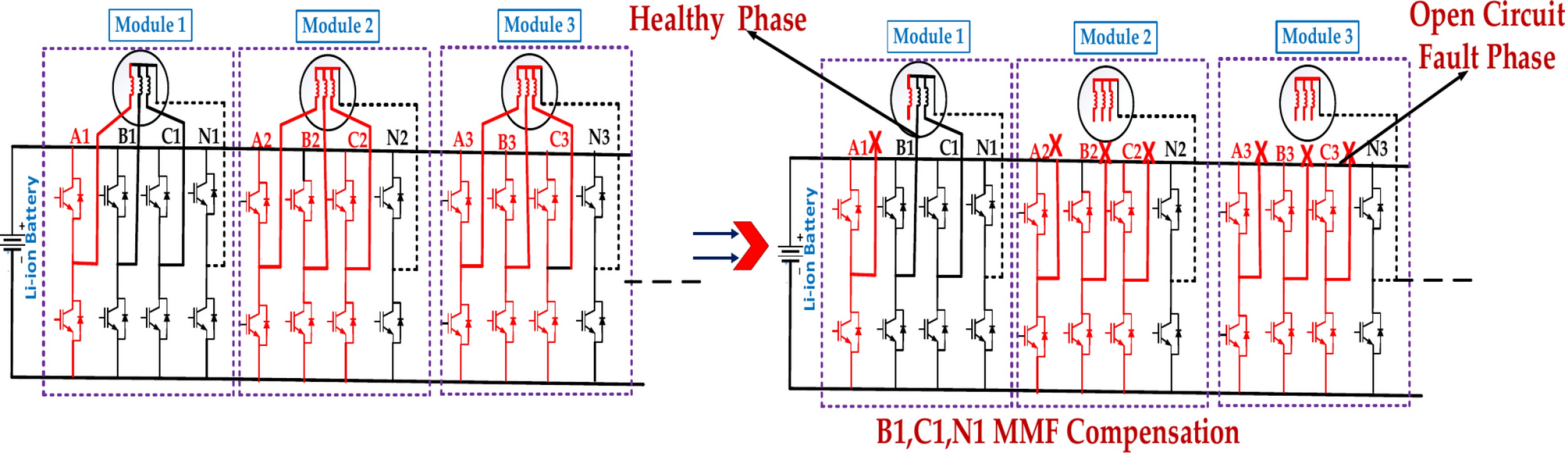
The first case is the structure of the faulty module under the extreme multiphase open-circuit fault.

Figure 11 represents the faulty module under the first case’s extreme multiphase open circuit fault. The exact location of the fault within the module will determine the extent of the damage and the required repair actions^46^. The effectiveness of the protection system in detecting and isolating the fault will impact the overall system reliability. The above diagram represents the C1, B1, and N1 MMF Compensation to B2 and C2 for the open circuit fault and healthy phases.

**Fig. 12:**
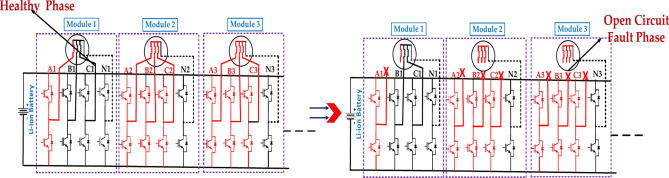
The structure of the faulty module under the extreme multiphase open-circuit fault is in the second case.

Figure 12 represents the fault module under Extreme open circuit faults in the second case. The exact location of the fault within the module will determine the extent of the damage and the required repair actions^47^. The effectiveness of the protection system in detecting and isolating the fault will impact the overall system reliability. The above diagram represents the open circuit fault phase and the healthy phase.

Table 9 summarizes the impact of different open-circuit faults on a system with two modules (Module I and Module II). Open-circuit faults significantly reduce the system’s torque capability. Depending on the specific fault and remaining healthy phases, different control strategies are employed to maintain some level of operation. Winding Reconstruction Product (WR) comes into play when the system loses torque capability but still has some rotating inertia. MMFC This stands for "Magneto-Motive Force Control," a technique used to maintain balanced motor operations^49^. FOC stands for "Field-Oriented Control, " another control method for optimizing motor performance. WR stands for "winding Reconstruction Product," a term representing the inertia of the rotating parts in the system.

**Table 9:** The multiphase open-circuit fault with the external open circuit fault tolerance circuit strategy^48^.

S. No	Description	(Without-fault)			(With-fault)			Torque capability	Control strategy
		Module-I	Module-II	Module-III	Module-I	Module-II	Module-III		
1	1-Phase open circuit fault	A1, B1, C1, N1	A2, B2, C2, N2	–	A1, B1, N1 (MMF compensation)	A2, B2, C2 (FOC)	–	(n-0.423)/n	MMFC
2	2-Phase open circuit fault (Case-i)	A1, B1, C1, N1	A2, B2, C2, N2	–	A2, B1, N1 (MMF compensation)	B2, C2, N2 (FOC)	–	(n-0.846)/n	MMFC + WR
3	2-Phase open circuit fault (Case-ii)	A1, B1, C1, N1	A2, B2, C2, N2	–	A1, B1, N1 (MMF compensation)	A2, B2, N2 (MMF compensation)	–	(n-0.846)/n	MMFC
4	3-Phase open circuit fault (Case-i)	A1, B1, C1, N1	A2, B2, C2, N2	–	A2, B1, C1 (field oriented control)		–	(n-1)/n	WR
5	3-Phase open circuit fault (Case-ii)	A1, B1, C1, N1	A2, B2, C2, N2	A3, B3, C3, N3	B1, C1, N1 (MMF compensation)	A3, B2, N2.(MMF compensation)	B3, C3, N3 (MMF compensation)	(n-1.269)/n	MMFC + WR
6	Multiphase open circuit fault (Case-i)	A1, B1, C1, N1	A2, B2, C2, N2	A3, B3, C3, N3	A1, B1, N1 (magneto motive force compensation)			(0.577)/n	MMFC + WR
7	Multiphase open circuit fault (Case-ii)	A1, B1, C1, N1	A2, B2, C2, N2	A3, B3, C3, N3	A2, B1, N1 (magneto motive force compensation)			(0.577)/n	MMFC

WR = Winding reconstruction; MMFC = magneto motive force compensation; FOC = field oriented control.

## Fault-tolerance inverter topologies for leg level techniques

Electric vehicles (EVs) use permanent magnet synchronous machines for various purposes. Electric propulsion is just one of these uses; additional electrical subsystems include active suspension, electric steering, brake-by-wire, and HVAC systems. Adding a high level of reliability to motor drives used for propulsion cannot be justified if a drive system malfunction does not substantially raise the risk of an accident or if a post-fault strategy is not financially viable for a particular vehicle model. The primary reason for this is that EV driving systems must be extremely dependable and safe. To guarantee the safety of the EVs, such drive systems require fault-tolerant control or FTC^50^. Furthermore, FTC is ideally suited to enhance electromechanical systems’ dependability, availability, and continuous operation following the most recent requirements to improve functional safety for automotive electric/electronic systems. Power switch failures prevent a three-phase motor drive system from producing a steady output speed. Fault-tolerant electric drive systems generally modify the inverter drive topology to enable uninterrupted speed production even during electrical failures.

**Fig. 13:**
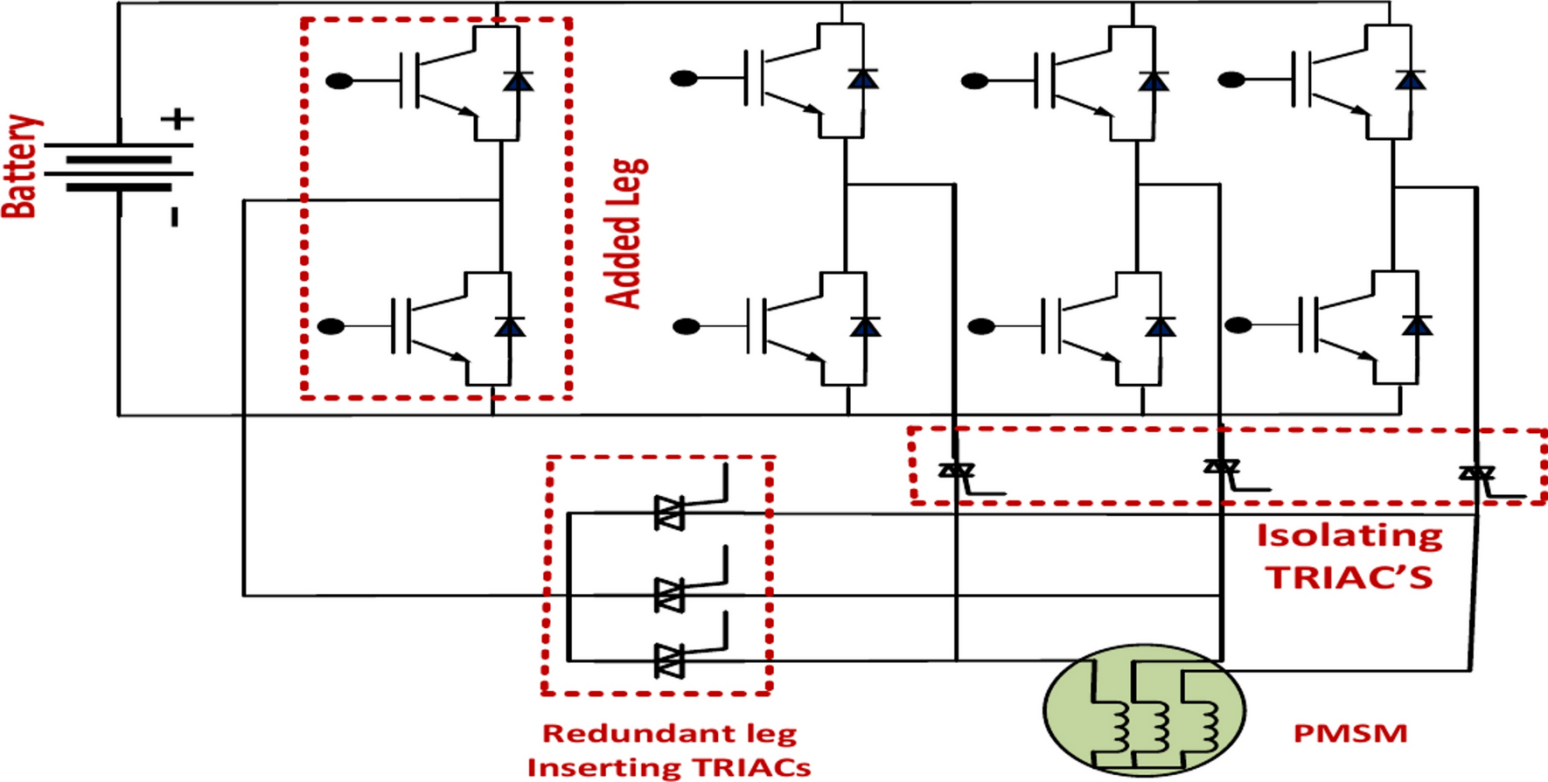
Fault-tolerance inverter topologies for leg level techniques.

Figure 13 represents the fault tolerance leg, an essential part of the system intended to improve its dependability and continue functioning despite failures. Typically, it comprises redundant power switches (diodes or transistors) that can take over a malfunctioning component. Figure 1 above illustrates the many methods that have been proposed in the literature to attain fault tolerance for 3-phase inverter motor drives. This Topology’s primary drawback is the increased number of TRAICS bypasses. In the fault-tolerance topology, a three-phase inverter’s bypass TRIACs are reduced from six to one. Another potential fault-tolerance converter design is an additional inverter leg that links to the machine’s neutral point via a bypass TRIAC^51^. The motor in this Topology can continue to run at its rated speed and torque even in the event of a fault. Nevertheless, the power switches will tolerate additional current, leading to overheating and uneven heat across them. Moreover, torque pulsation may result from this Topology since it gives the current third harmonics a path.

**Fig. 14:**
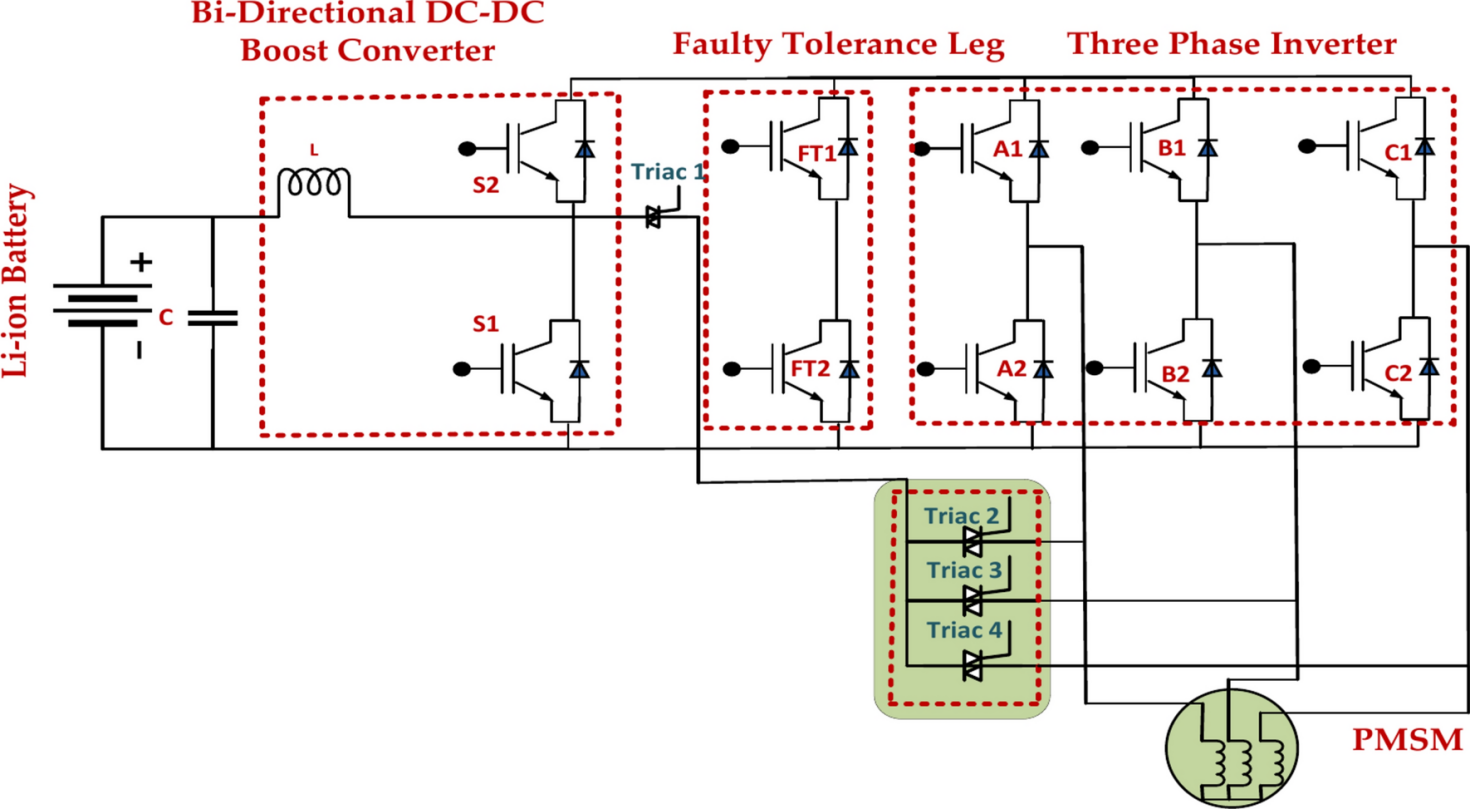
Fault-tolerant topology of an electric vehicle power conversion system using leg-level techniques.

The enhanced fault-tolerant Topology and control shown for the power conversion system utilized in the EV propulsion system are shown in Fig. 14. The Topology provides combined FTC for the DC–DC boost converter and 3-Phase inverter by utilizing an extra inverter leg and four bypass TRIACs. The FTC used in this paper is for power conversion problems involving open circuit switches. The diagram uses more power switches (T1, T2, T3, and T4) and diodes to implement the fault tolerance leg. A malfunctioning power switch can be avoided by turning on these parts, and the inverter can continue functioning. When the system continuously checks on the condition of every component, it is said to be fault-detecting^52^. The fault tolerance mechanism starts when a problem is identified, like an open or short circuit in a power switch. Redundancy Switching: The fault tolerance system transfers the load to the redundant component from the defective component. This is usually accomplished using bypass diodes or other switching devices. Continued Operation: The system can continue functioning even with a decreased capacity or performance since the redundant component has taken over. Fault tolerance legs raise the system’s overall availability while improved system availability reduces downtime. Lower Maintenance Expenses Fault tolerance legs can lower maintenance costs by prolonging the life of components. Enhanced Security: Fault tolerance legs can be used in safety–critical applications to assist in guaranteeing safe operation and avoiding catastrophic failures.

## Lithium-ion battery faulty conditions

Table 10 overviews common lithium-ion faults, their causes, and results. Now, they are typically detected by the battery management system (BMS). The specific causes, consequences, and detection methods for lithium-ion battery faults can vary depending on the specific battery chemistry, design, and operating conditions. By understanding these common faults and their potential consequences, engineers and users can take necessary precautions to ensure lithium-ion batteries’ safe and reliable operation.

**Table 10:** Lithium-ion battery different fault conditions^53^.

Lithium-ion battery fault				
Fault	Reason	What happens?	Results	How is BMS detected?
Internal short fault	1. The insulating layer between the electrodes falls	1. Cause the lithium ions and electrode to be released at the anode and travel across the electrolyte towards the cathode	1. Triggers contact between the anode and the cathode	1. Incorrect voltage and current measurement
			2. Thermal runaway	2. Inaccurate SOC estimation from the BMS
			3. High-capacity cells are at a higher risk of thermal runaway	
External short circuit	1. A low-resistance path connects tabs	1. External heat-conducing element makes contact with positive and negative terminals	1. Excessive discharge of the energy that is being stored in a cell	1. Incorrect voltage and current measurement
	2. Electrolyte leakage from cell swelling due to gas generation during overcharge	2. Electrical connection between the electrode		2. Inaccurate SOC estimation from the BMS
	3. Water immersion and collision deformation			
Overheating	1. A low-resistance path connects tabs	1. External heat-conducting elements make contact with the positive and negative terminals	1. Excessive discharge of the energy that is being storage in a cell	1. Incorrect voltage and current measurement
	2. Electrolyte leakage from cell swelling due to gas	2. Electrical connection between the electrode		2. Inaccurate SOC estimation from the BMS
	3. Generation from side reactions during overcharge			
Thermal run away	1. Charging of the battery under extreme charging currents and high temperatures restricted air circulation	1. The battery heats up and explodes	1. Battery burst	1. Physical verification
		2. Increase the number of Charge/discharge cycles		
Sensor fault	1. Vibration, collision electrolyte leakage, and other physical factors Loose battery terminals or corrosion around the battery sensor	1. Accelerate the degradation process of a battery	1. Inefficient thermal management	1. Through sensor
		2. Delay the BMS functions due to incorrect state estimation	2. Inaccuracy in the BMS decreases in battery life	
		3. Cause incorrect measurements to the BMS	3. Overheating ageing under high temperatures	
		4. Entire pack and lower to exceed the upper and lower voltage limits	4. Inaccurate SOC and SOH estimation	
Cooling system fault	1. Cooling motor or fan falls to operate	1. Server fault as it leads to a direct failure of the battery	1. Overheating	1. Through sensors
	2. Outdated fan wiring, fault temperature sensor, or a broken fuse		2. Thermal runaway	
	3. Temperature sensor and cooling system faults can’t be spared from each other as they both depend on the temperature range			
Cell Connection fault	1. Poor electrical between the cell terminals, the terminal may become loose from vibration or corroded by impurities over time	1. The cell resistance can increase drastically, leading to cell imbalance due to uneven current	1. External short circuit	1. Through sensors
		2. Overheating of the faulty cell	2. Thermal runaway	

### Internal and external lithium ion battery and causes

**Fig. 15:**
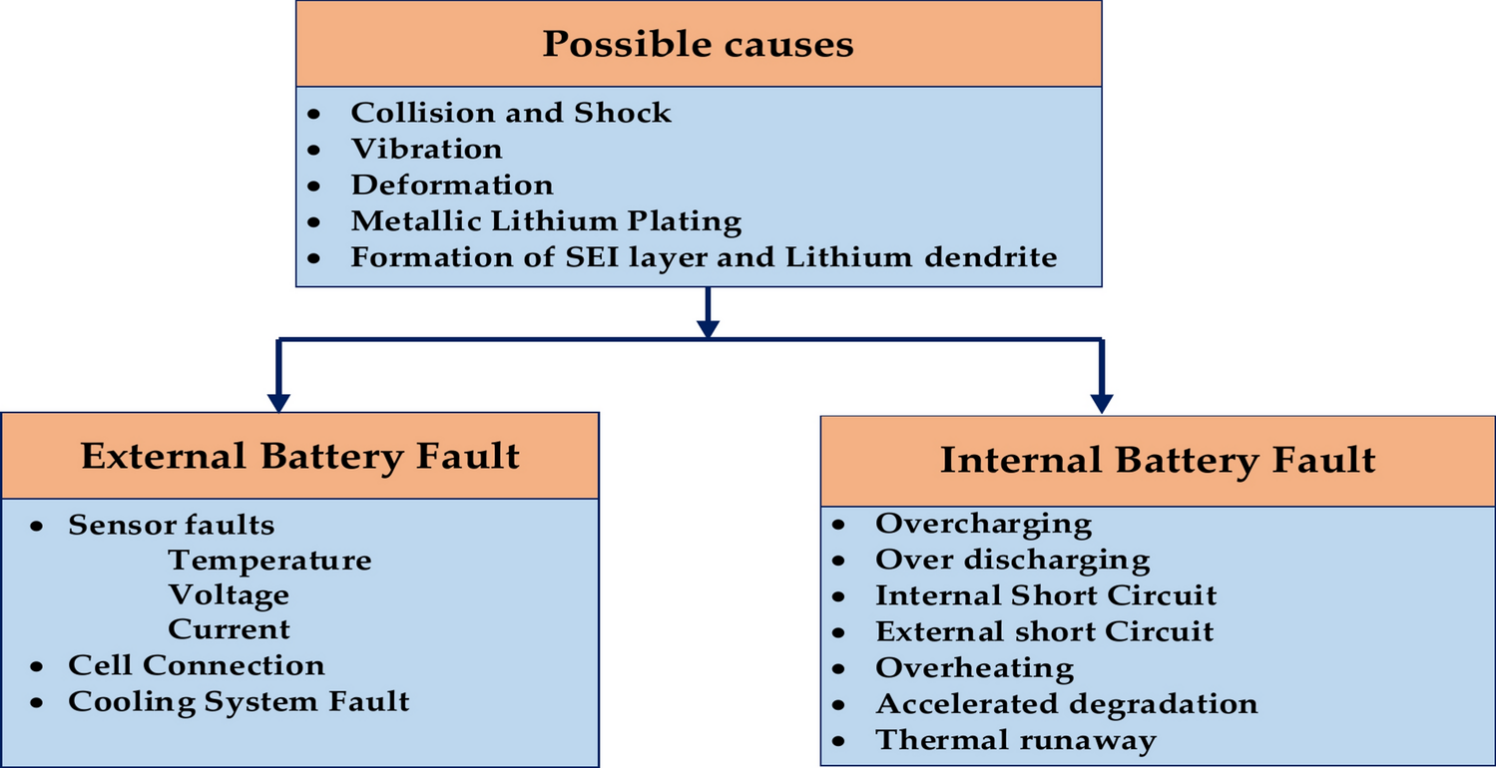
Internal and external ion battery causes.

The diagram uses two primary categories to classify possible battery issues: Battery defects might be internal or external. Figure 15 represents the flowchart outlining potential causes for battery faults, categorised into external and internal faults. This provides a helpful overview of the different types of battery faults and their potential causes. It is important to note that these are not all-inclusive lists; other faults can also occur.

#### External fault

Sensor faults are an external problem. They might result in inaccurate readings from temperature, voltage, or current sensors, which can misinterpret battery conditions^54^. Cell Connection Issues: Inadequate connections between cells may cause an uneven voltage distribution and perhaps lead to overheating. Cooling System Issues: Overheating and a shorter battery life might result from cooling system issues. Coding System Errors: Software errors in the battery management system may result in incorrect charging or discharging cycles.

Table 11 represents the Comparison of external battery fault Diagnosis techniques. While the focus seems to be on sensor faults, using State of Charge (SOC) and temperature parameters suggests the potential for detecting other faults like cell imbalance or temperature anomalies. Employs the Extended Kalman Filter (EKF), a powerful statistical estimation technique, for fault detection and estimation. The ability of each technique to detect and isolate a wide range of faults should be thoroughly investigated. This analysis provides a general comparison of the presented techniques. A more in-depth evaluation would require a detailed review of the original research papers and their experimental results.

**Table 11:** Comparison of external battery fault diagnosis.

Author	Comparison of external battery fault diagnosis				Achievements
	Citations	Fault parameters	Faults in battery	Technique	
Xia et al.	^55^	Voltage measurement	Detecting sensor and cell faults	Fault-tolerant voltage monitoring technique for a series-connected battery pack	Sensor faults can be isolated without any hardware setup
Lombardi et al.	^56,57^	Relationship between voltage sensor measurement and current sensor measurement	Identifying the sensor faults of Lithium-ion battery	Kirchhoff’s law	Finished lithium-ion battery fault detection and isolation of current and voltage sensors
Liu et al.	^58–60^	Residues from the structural analysis theory generated based on the EKF method	Detecting the sensor faults	Structural analysis theory	Used for reducing the noise but also increased the computational cost
Liu et al.	^61^	Fault parameters from SOC, temperature, and voltage	Estimating the output voltage of faulty voltage	EKF	Used for reducing the noise but also increased the computational cost

#### Internal fault

Overcharge and over-discharge are internal faults that surpass the battery’s charge or discharge limits and potentially harm the cells. Internal Short Circuit: A cell’s anode and cathode may be directly connected electrically, which could cause a thermal runaway and a quick release of energy^62^. An external short circuit between the battery terminals may result in excessive current flow and overheating. Overheating in elevated temperatures can hasten the deterioration and aging of battery cells^63^. Repeated cycles of charging and draining, particularly at high temperatures, can hasten battery damage. A thermal runaway is a self-sustaining chain reaction that produces heat and causes additional overheating, ultimately resulting in a catastrophic failure^64^. By identifying the possible reasons for battery malfunctions and implementing suitable preventative measures, battery-powered systems’ dependability and security can be greatly increased.

Table 12 represents the comparison of internal battery fault diagnosis. Overcharge and over-discharge are internal faults that surpass the battery’s charge or discharge limits and potentially harm the cells. Internal Short Circuit: A cell’s anode and cathode may be directly connected electrically, which could cause a thermal runaway and a quick release of energy. Excessive current flow and overheating may result from an external short circuit between the battery terminals. Overheating: High temperatures can accelerate the aging and degradation of the battery cells. Battery degeneration can be accelerated by frequent cycles of charging and draining, particularly when temperatures are high. A self-sustaining chain reaction that generates heat causes additional overheating, resulting in a catastrophic failure known as a thermal runaway. Battery-powered systems can be made far more reliable and safer by identifying the possible causes of battery failures and implementing suitable mitigation techniques.

**Table 12:** Comparison of internal battery fault diagnosis.

Author	Comparison of internal battery fault diagnosis				
	Citations	Fault parameters	Faults in battery	Technique	Achievements
Amardeep et al	^65^	Battery model parameters	Detecting overcharge and over-discharge faults	Extended Kalman filter	This model efficiently detects the overcharge and over-discharge faults in real-time
Yang et al	^66^	Fault parameters for abnormal voltages	Identifying the Li-ion battery faults	Artificial Neural Networks (ANN)	A complete battery fault diagnostics model for abnormal voltages is built based on extensive data regulation
Jing et al	^67^	Increasing temperature and decreasing voltage	Detecting overcharge faults	Ruled-based method	This model is efficient in detecting overcharge faults and alerts the users early
Vinay et al	^68^	Fault parameters from Soc, temperature, and voltage	Li-ion battery overcharging and over-discharging	Fuzzy logic	This approach efficiently and precisely identifies overcharge and over-discharge issues in Li-ion batteries

### Fault code-CAN communication

**Fig. 16:**
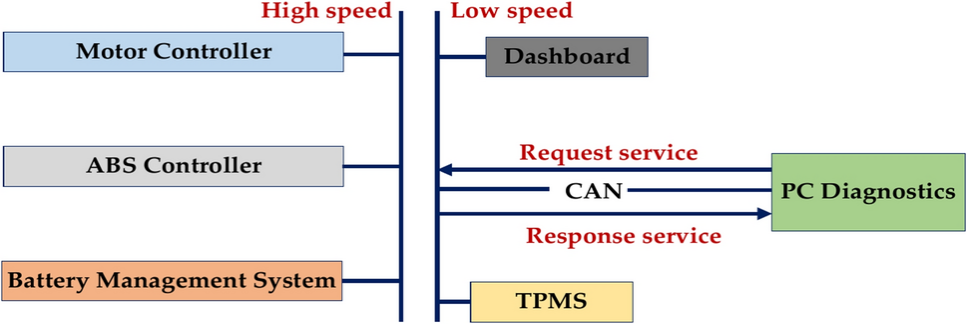
Fault code CAN communication protocol.

Figure 16 represents the several ways that (Controller Area Network) communication failures might appear, and certain fault codes frequently denote them. The following list of common CAN communication trouble codes includes possible explanations. Common Reasons for Control Area Network Communication Errors When the wire is loose, damaged, or broken, it can interfere with the CAN bus signal. Additionally, the wire may be impacted by corrosion or water damage. Broken Connectors: Corroded, loose, or broken connections may make it impossible for modules to communicate properly. Communication issues might arise from a malfunctioning module that sends the wrong signals or doesn’t react to requests. Other electronic devices’ electromagnetic interference (EMI) can interfere with the CAN bus signal. Problems with the power supply Modules and the CAN bus may not function properly if the power source is inadequate or unreliable. Malfunctioning Module is a faulty module that can send incorrect signals or fail to respond to requests, causing communication errors. Interference is electromagnetic interference (EMI) from other electronic devices that can disrupt the CAN bus signal. Power Supply Issues An insufficient or unstable power supply can affect the operation of modules and the CAN bus.

### Diagnostic trouble code (DTC)

Table 13 represents the car’s onboard diagnostic (OBD) system using diagnostic trouble codes (DTCs) to find and diagnose particular problems. When the car’s computer notices a change from standard operating conditions, these codes are frequently activated. Each of the five characters that make up a DTC code represents a distinct piece of information regarding the issue.

**P**: Powertrain (engine, transmission, fuel system, etc.)
**C**: Chassis (brakes, steering, suspension, etc.)
**B**: Body (interior components, lighting, etc.)
**U**: Network and control modules

**Table 13:** Diagnostic trouble code.

P1100	Indicates that the battery management system module voltage is low
P1101	Indicates that the BMS module voltage is too high
P1001	Indicates that the motor controller temperature is too high
P1010	Indicates that the motor voltage is too low
P1021	Indicates that the motor temperature is too high

DTC codes are only the beginning of a diagnosis. A skilled technician should always be consulted for accurate diagnosis and repair. Maintain your car regularly to avoid possible problems. By being aware of DTC codes, you can better assess the condition of your vehicle and take quick action to fix any issues.

Table 14 represents the number divided into two parts, likely representing a 16-bit value. The “High Byte” represents the more significant bits (left side), and the “Low Byte” represents the less significant bits (right side). To convert the binary number to decimal, you would typically use the place value system, where each bit position has a weight of 2 raised to the power of its position (starting from 0 for the rightmost bit).

**Table 14:**
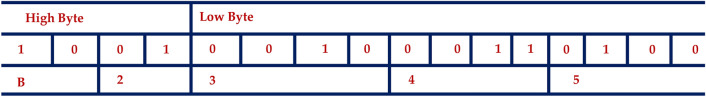
Binary representation of a number for high byte and low byte.

#### Lithium-ion battery fault diagnosis algorithm

**Fig. 17:**
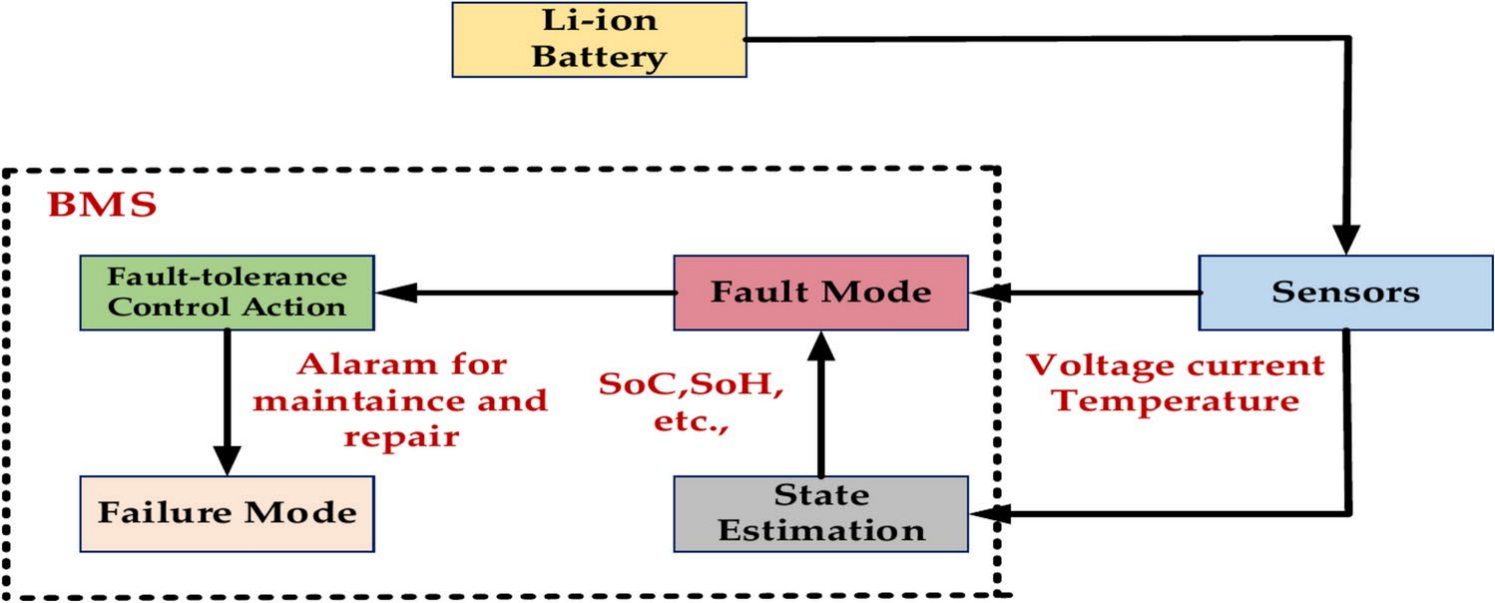
Lithium-ion battery fault diagnosis algorithm.

Figure 17 represents the essential algorithm to determine the primary failure modes and the sensor data that may be used for diagnostics. Rapid temperature rise and even thermal Runaway can result from an internal short circuit (ISC), which is a direct electrical connection between the anode and cathode. A battery’s capacity to hold charge gradually deteriorates over time. Increased internal resistance that impacts battery efficiency and performance is known as increased internal resistance. Exposure to extremely high or low temperatures can cause rapid deterioration and possible safety hazards. This is known as thermal abuse. It is feasible to create efficient fault detection algorithms that can guarantee these vital components’ secure and dependable operation by integrating model-based and data-driven methodologies and considering the unique properties of lithium-ion batteries.

**Fig. 18:**
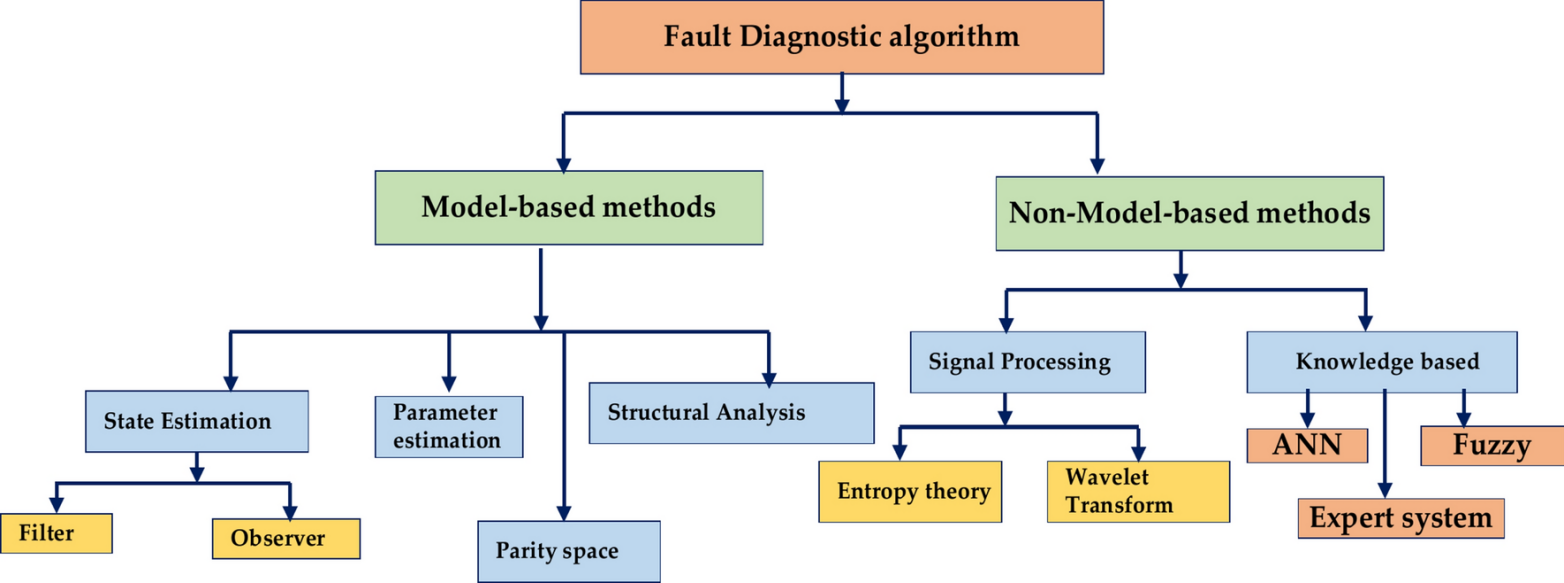
Fault diagnostic algorithm.

Figure 18 represents the essential step in guaranteeing the dependability and security of intricate systems: fault identification. Corrective action entails recognizing, detecting, and isolating errors. The two main methods for diagnosing faults are model-based and non-model-based. Model-based fault diagnostics uses mathematical models of the system to forecast how it will behave in normal and malfunctioning circumstances. Deviations from the intended behavior might be recognized as possible flaws by comparing them to the actual observed behavior. On-model-based fault diagnosis systems use statistical and machine-learning techniques to evaluate past data and find patterns linked to failures.

Table 15 represents the Model, and non-model-based methods provide a strong theoretical foundation but can be complex and computationally expensive. Non-model-based methods rely heavily on data and can achieve high accuracy but require significant data collection and may have limitations in terms of interpretability. It’s important to note that a combination of model-based and non-model-based approaches can often lead to more robust and effective fault diagnosis systems.

**Table 15:** Model and non-model-based method.

Model-based methods	1. Electrochemical, electrical, thermal, and combinations of interdisciplinary models
	2. Model-based methods are often used in fault diagnosis for their simplicity and cost-efficiency
	3. Model-based methods include state estimation, parameter estimation, parity equation, and structural analysis
	4. Impedance spectroscopy, Kalam filters, multi-model adaptive estimation (MMAE) technique, recursive least squares (RLS), energy balance equation (EBE)model
Non-model-based methods	1. We primarily rely on battery data collection, although we are still using battery modelling to an extent
	2. They can improve fault diagnostics. Accuracy but might require the amount of fault data
	3. Very high computational cost, which is impractical for usage in the BMS

#### General fault detection algorithm

**Fig. 19:**
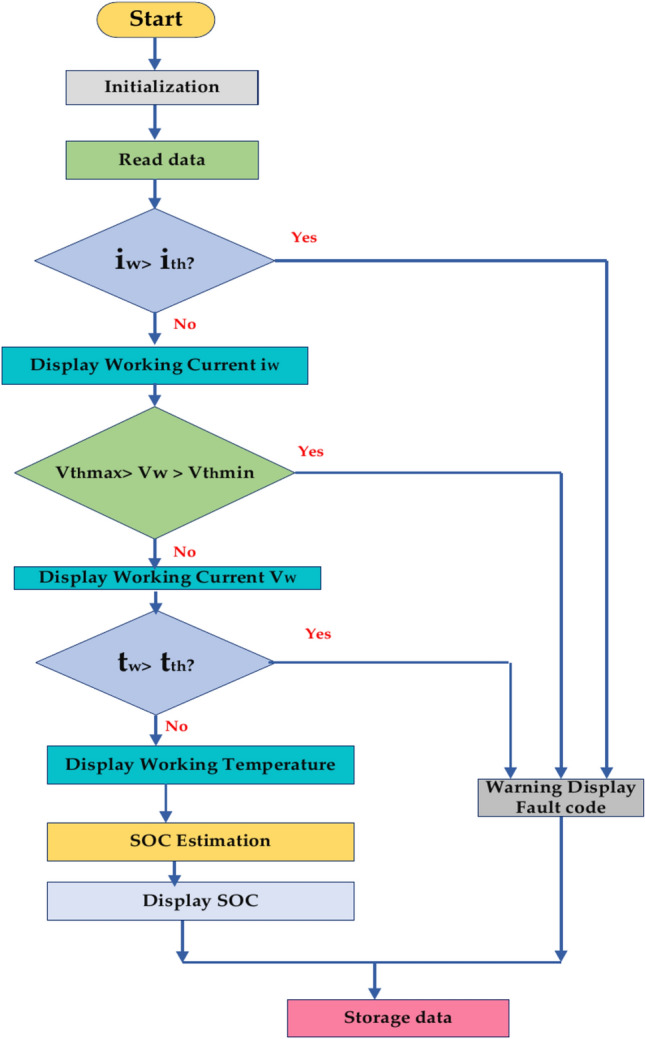
General fault detection algorithm.

Figure 19 represents the general fault detection algorithm for the Battery Management System (BMS), which is shown in a simplified form in the flowchart. It lists the crucial actions to monitor and control the battery’s condition and functionality. Start the system and set up all the required settings and variables. Configuring the BMS parameters and establishing communication with the battery comprise the initialization stage. Read Data: the BMS retrieves vital battery data, such as temperature, voltage, current, and state of charge (SOC). The system keeps an eye on the current draw to ensure that it stays within the specified limits. Let’s say it exceeds the highest limit. It generates a problem or warning code. Monitoring Voltage^69^. The system checks to see if the battery voltage is within a safe range. The proper measures are implemented if it’s too high or too low. Temperature Monitoring: The system determines whether the battery’s temperature falls within the acceptable range for safe operation. A warning is generated, or cooling devices may be triggered if the warning is too high. SOC Estimation: Using the measured voltage, current, and temperature, the BMS calculates the battery’s remaining capacity. Show the BMS, a user interface that shows the voltage, temperature, working current, and predicted state of charge. Warning Display: A warning message or fault code is shown to the user if a fault situation is found. Storage Information: For analysis, logging, or future use, the BMS may keep the gathered information^45^.

## Open circuit switch fault-tolerance in five-level inverter

### Open circuit fault S1

An open circuit problem in switch S1 in the diagram below would indicate that switch S1 cannot conduct current. Voltage imbalance is the voltage distribution across the DC link capacitors that may develop out of balance, which can strain the parts and cause damage. The inverter’s output voltage capability is decreased when one of the switching devices that produces the higher voltage levels malfunctions. This is known as lower output voltage. A decrease in the number of active switching states may result in a higher level of harmonic distortion in the output waveform. Overcurrent in other switches: the remaining switches may encounter increased current strains, which could result in an early failure. Protection System Activation is the process by which the inverter’s protection system may identify a problem and initiate a shutdown in order to stop additional harm^70^. The switches, diodes, inductors, capacitors, and transformers are among the several parts of the inverter depicted in the diagram. The control system utilized to operate the inverter is also displayed.

**Fig. 20:**
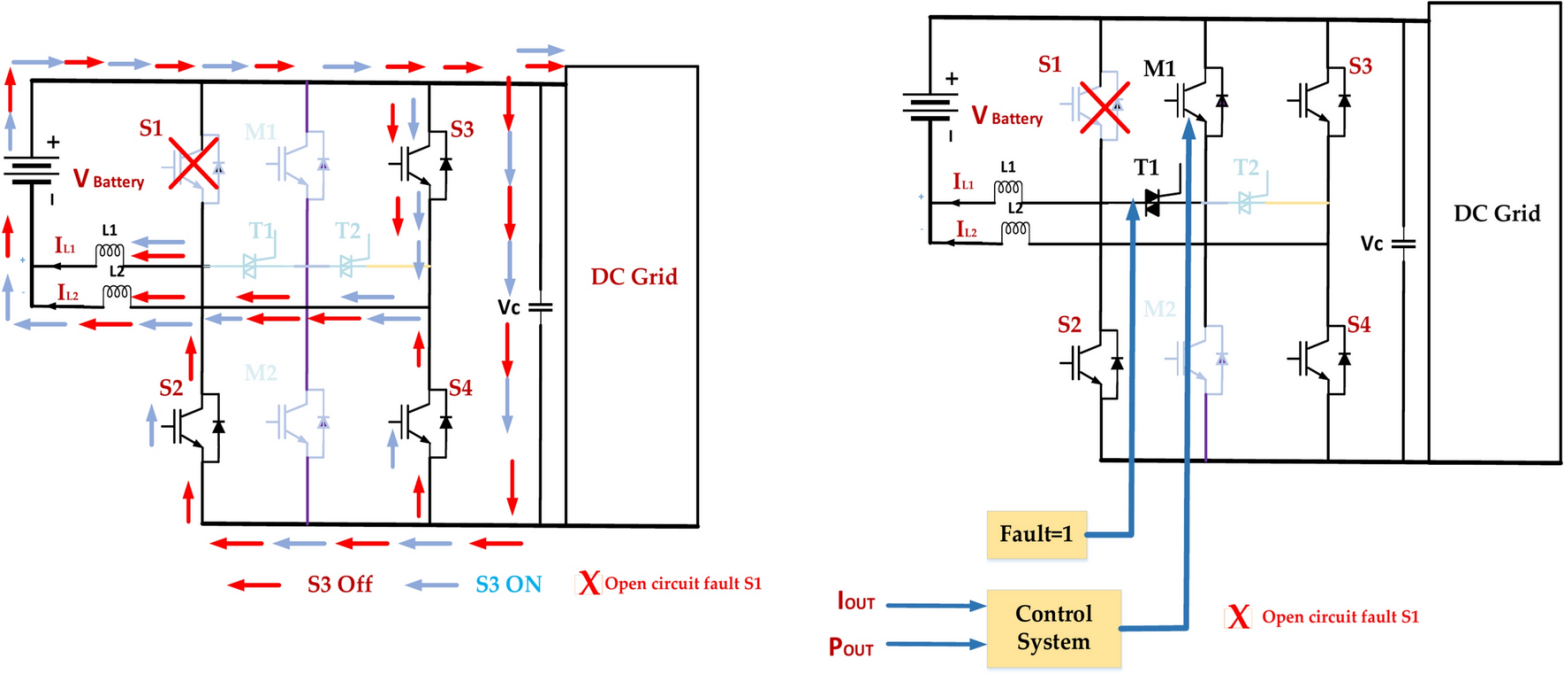
Open circuit inverter fault tolerance in switch S1.

Figure 20 represents the open circuit inverter’s fault tolerance feature implemented using a control system that monitors the status of the inverter’s components. If a component fails, the control system automatically switches the inverter to a backup configuration that does not use the failed component. This allows the inverter to continue to operate even if one of its components fails^71^. The Five-level Active Neutral Point Clamped Inverter (ANPC) fault Tolerance inverter is a complex device, but it is an important technology used in various applications. It is a reliable and efficient way to convert DC power into AC power.

### Open circuit fault S3

**Fig. 21:**
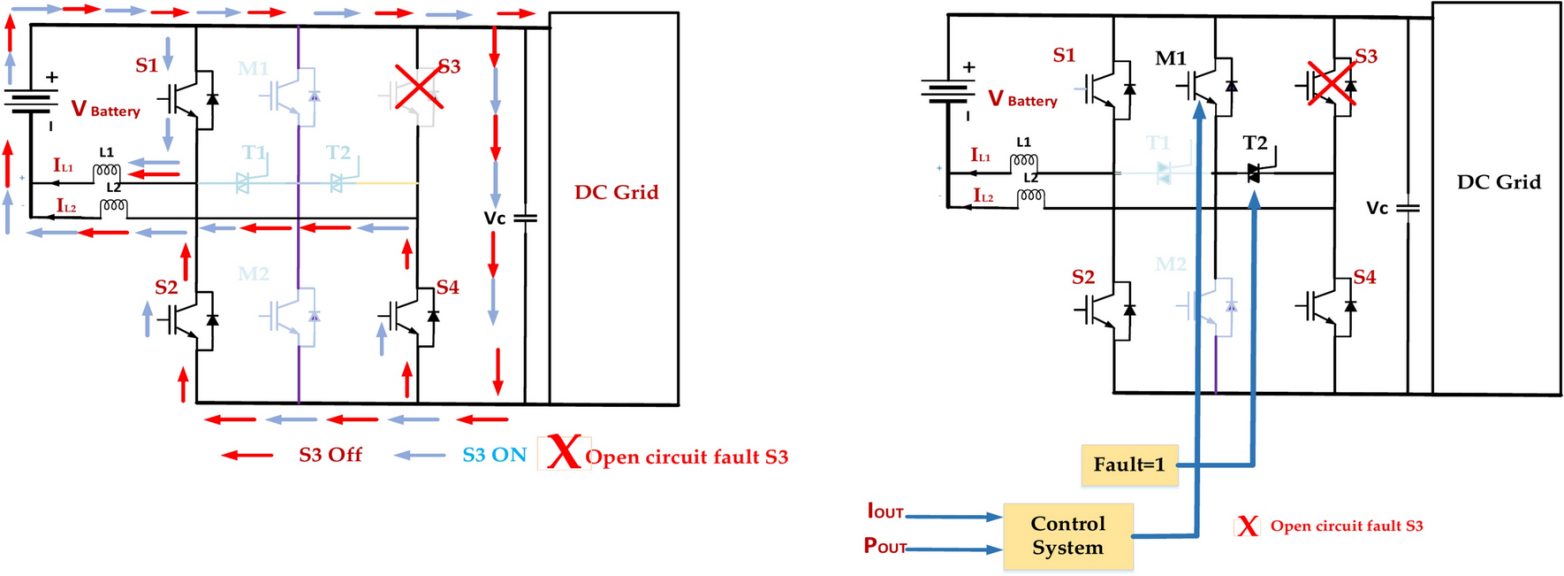
Open circuit inverter fault tolerance in switch S3.

Fault Detection and Isolation implement a robust fault detection system to quickly identify open circuit faults in S3, as presented in Fig. 21. The faulty leg is isolated to prevent further damage and maintain the operation of the remaining healthy legs. Redundancy incorporates redundant switches or modules to provide backup functionality in case of failures^72^. This can be achieved through parallel or series redundancy configurations.

Adaptive Control is Developing control strategies that can adapt to the fault condition by adjusting the switching patterns to minimize the impact on the output voltage and current waveforms. Overcurrent Protection employs overcurrent protection circuits to limit the current flowing through the remaining switches and prevent them from overheating^73^. The Monitoring and diagnostics department continuously monitors the health of the inverter components and implements diagnostic tools to identify potential issues before they lead to catastrophic failures.

### Open circuit fault S2

In the diagram of a 5-level ANPC inverter, an open circuit fault in switch S2 means that the switch fails to conduct current. This can have significant implications for the inverter’s operation. Voltage imbalance: the DC link voltage distribution may become unbalanced, potentially leading to overvoltage or undervoltage conditions on specific capacitors. The reduced output voltage will limit the inverter’s output voltage capability. The highest voltage level that can be achieved will be reduced, affecting the overall performance. Harmonic Distortion: The output voltage waveform will be distorted due to the reduced number of switching states available.

**Fig. 22:**
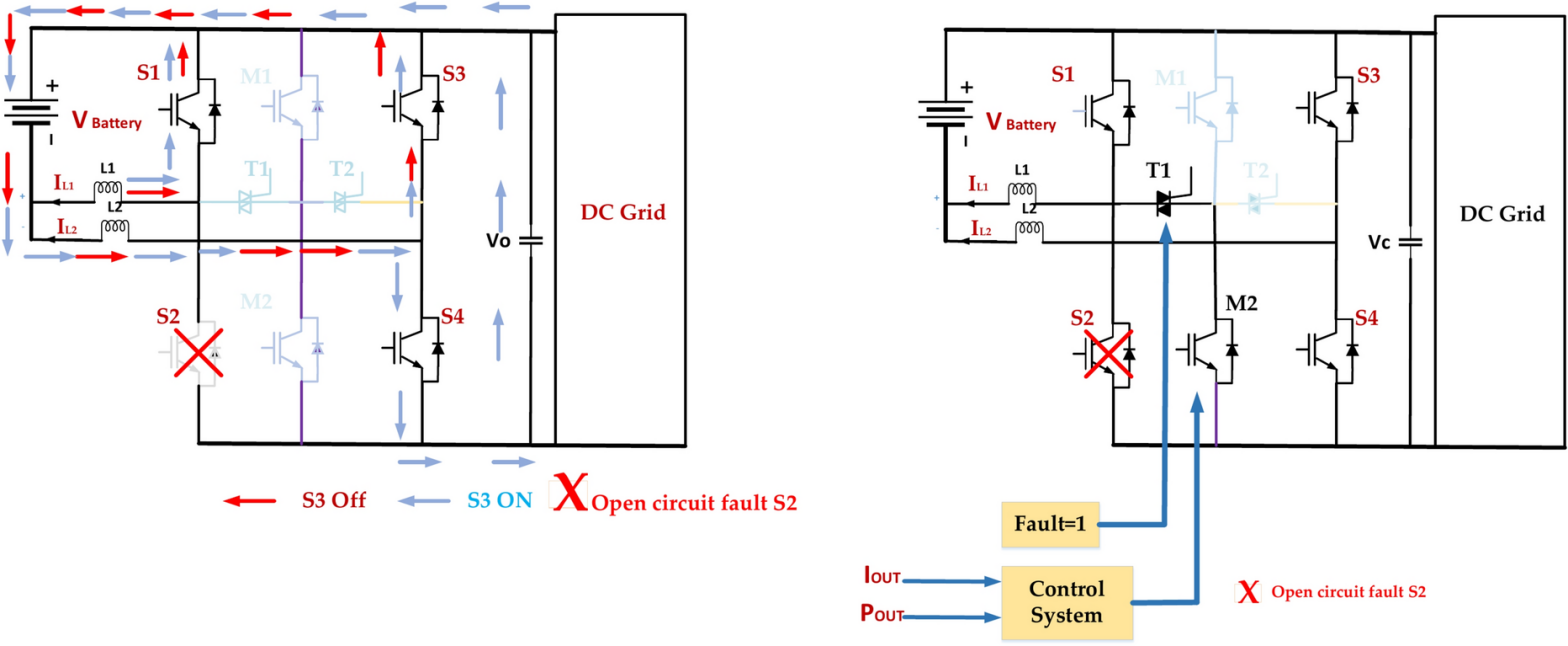
Open circuit inverter fault tolerance in switch S2.

Figure 22 represents this, which can lead to increased harmonic content in the output current and voltage. Increased Stress on Other Switches: The remaining switches may experience higher current and voltage stresses, potentially leading to premature failure. Protection System Activation^74^. The inverter’s protection system may detect the fault and trigger a shutdown to prevent further damage. Implement a robust detection system to identify the open circuit fault in S2 quickly. Isolate the faulty leg to prevent further damage and maintain the operation of the remaining healthy legs. By understanding the potential consequences of an open circuit fault in S2 and implementing appropriate mitigation strategies, it is possible to maintain the reliability and performance of the ANPC inverter.

### Open circuit fault S4

**Fig. 23:**
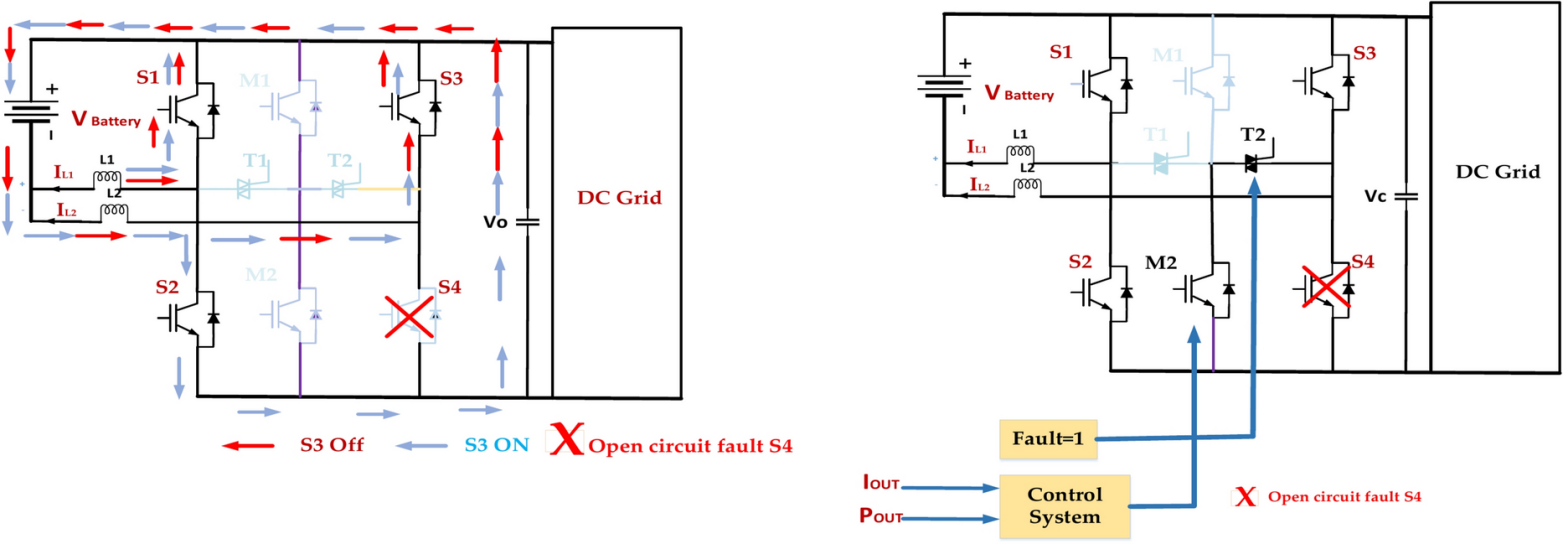
Open circuit inverter fault tolerance in switch S4.

Figure 23 represents the diagram provided of a 5-level ANPC inverter, and an open circuit fault in switch S4 means that the switch fails to conduct current. This can have significant implications for the inverter’s operation. The specific impact of the fault will depend on the inverter’s operating mode and load conditions. The effectiveness of the mitigation strategies will depend on the design of the inverter, the control system, and the protection mechanisms. It is important to have a comprehensive fault diagnosis and recovery system to minimize the fault’s impact and ensure the inverter’s reliable operation^75^. Understanding the potential consequences of an open circuit fault in S4 and implementing appropriate mitigation strategies can maintain the ANPC inverter’s reliability and performance.

**Fig. 24:**
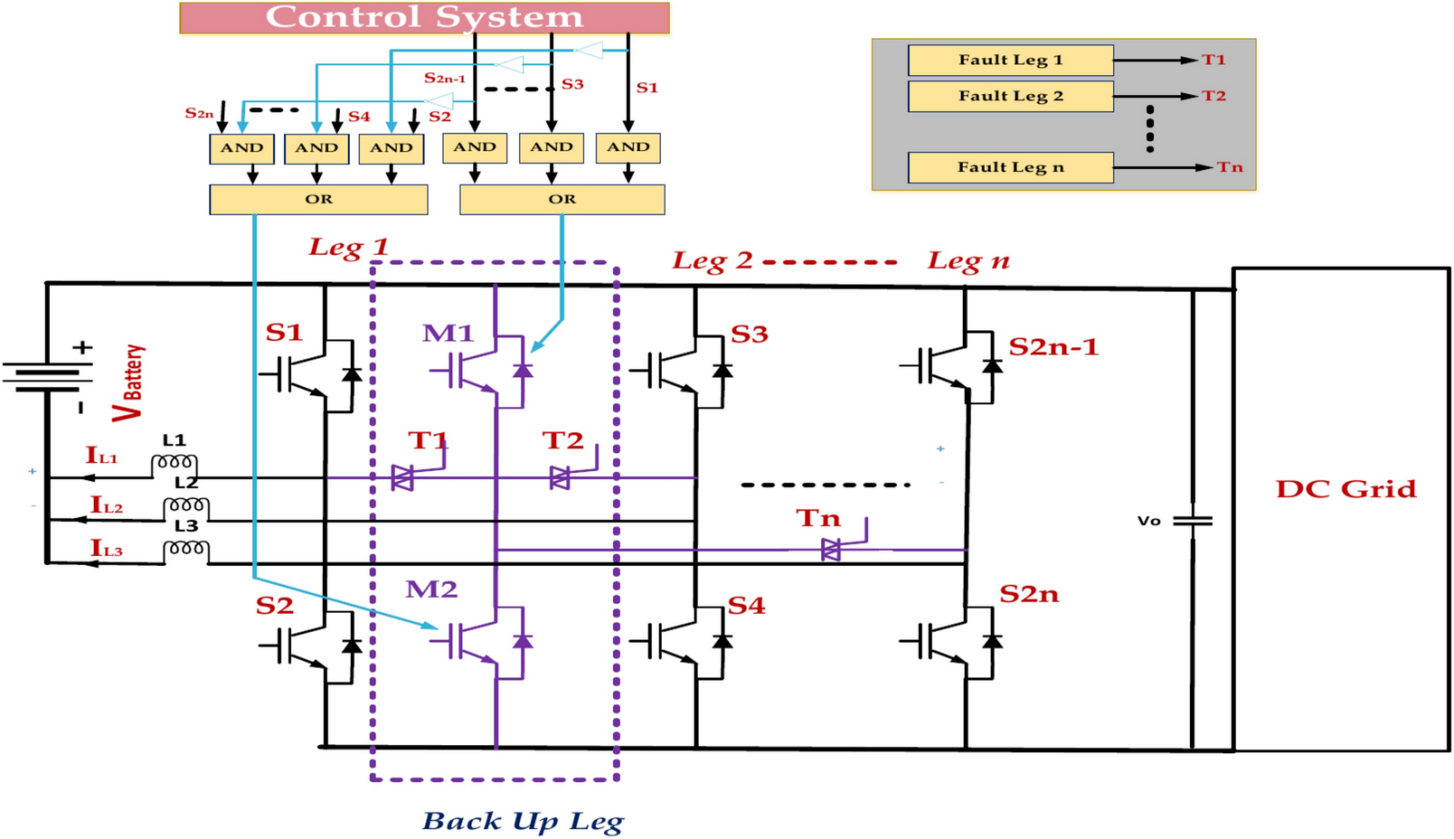
Analysing the fault tolerance mechanism in the five-leg multilevel inverter.

Figure 24 represents the fault tolerance achieved using redundant power switches (S_1_, S_2_, …, S_2n − 1_) and diodes (D_1_, D_2_, …, D_n_). Each leg of the inverter has a backup switch and diode that can take over in case of a fault. Fault Detections: the system continuously monitors the status of all power switches and diodes. If a fault is detected, such as an open-circuit or short-circuit, the fault detection mechanism triggers the fault tolerance mechanism. Redundancy Switching^76^. The fault tolerance mechanism switches the load from faulty to redundant components. This is typically achieved by controlling the gate signals of the switches. Continued Operation: With the redundant component taking over, the inverter can continue to operate, albeit with reduced capacity or performance^77^. Using fault tolerance principles and implementing appropriate techniques can significantly enhance the reliability and performance of multilevel inverter systems.

## Simulation results

**Fig. 25:**
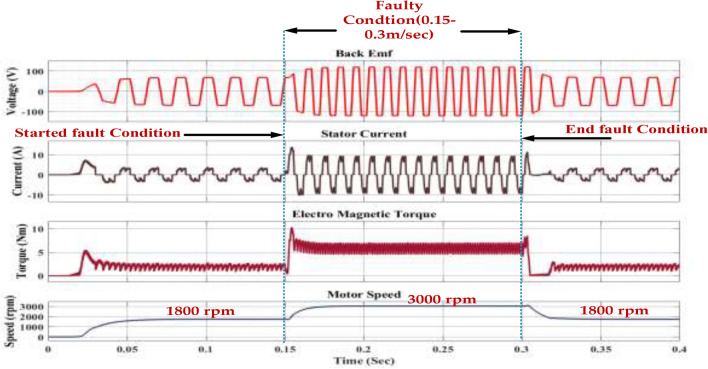
Wave forms of the motor speed, stator current of the PMSM under healthy mode, and faulty mode.

The dynamic response of a motor under a fault condition. It shows the variations in key parameters like voltage, stator current, electromagnetic torque, and motor speed over time. The fault is assumed to occur at 0.15 s and last for 0.3 s. The voltage waveform appears to be sinusoidal, indicating a three-phase AC supply. The voltage magnitude remains relatively constant throughout the simulation. The current waveform shows a significant increase during the fault period presented in Fig. 25.

This is likely due to increased impedance in the motor windings caused by the fault. After the fault is cleared, the current returns to its pre-fault level. The Electromagnetic torque initially increases as the motor accelerates. During the fault, the torque drops due to the increased impedance. After the fault is cleared, the torque recovers, and the motor regains its steady-state speed. The motor speed increases initially and then stabilizes at 3000 rpm. During the fault, the speed drops due to the reduced torque. After the fault is cleared, the motor accelerates back to its original speed. The impact of a fault on the performance of an induction motor. The fault causes a temporary drop in torque and speed, but the motor can recover after the fault is cleared. This analysis can be useful for understanding the behavior of induction motors under fault conditions and for developing fault detection and protection strategies.

**Fig. 26:**
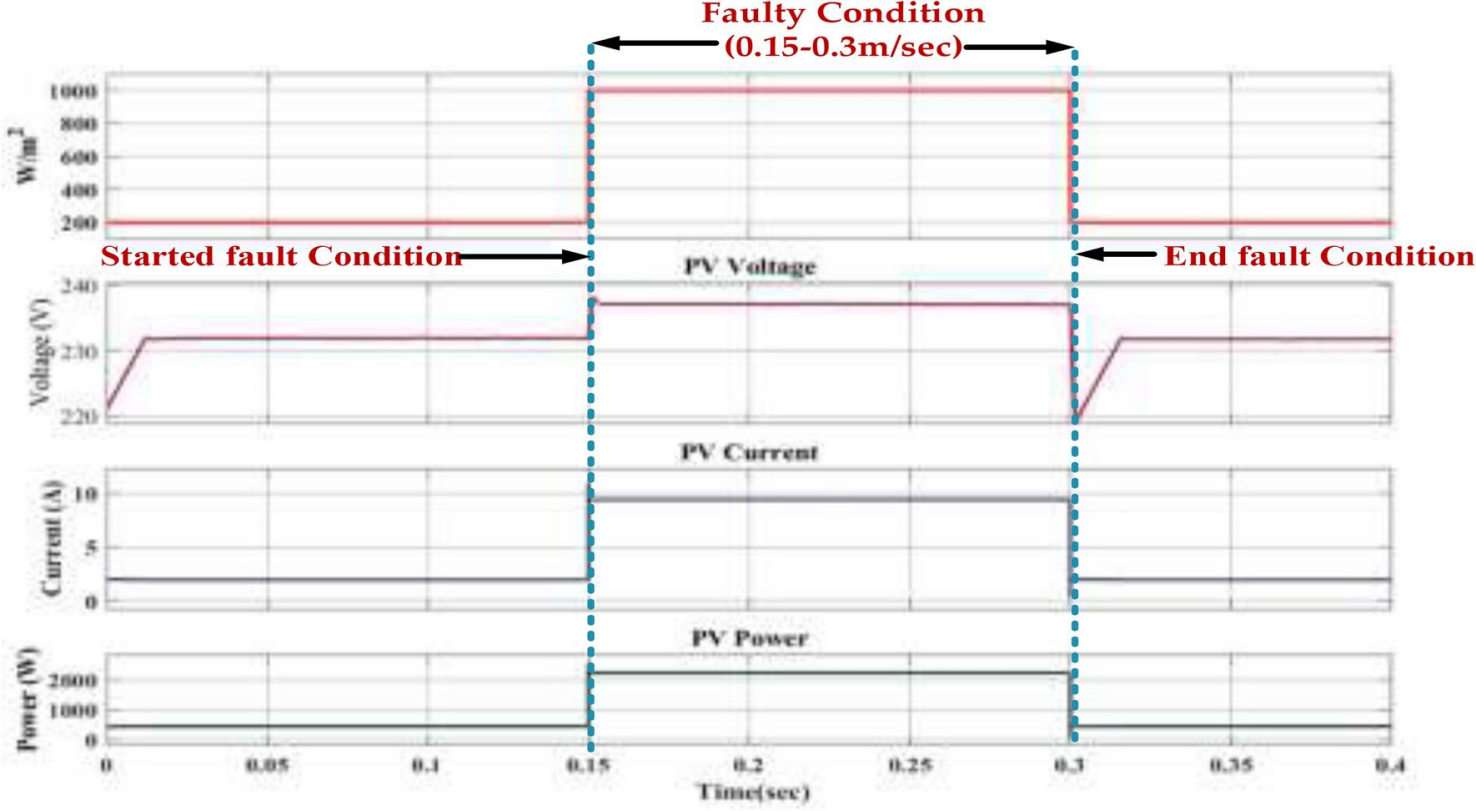
Wave forms of the PV voltage, current, power, and irradiation under healthy mode and faulty mode.

Figure 26 represents the fault occurs between 0.15 and 0.3 s. During this period, we observe the following changes in the system. The power output drops significantly to increase. This indicates that the fault has completely disrupted the power generation process. The voltage also falls to a low level, consistent with the absence of power generation. The current drops to zero, further confirming that the fault has halted the power flow. A short circuit in the PV module or the wiring could have resulted in a sudden drop in voltage and current, leading to zero power output. An open circuit in the PV module or wiring could also have caused a similar effect. If the PV system is connected to the grid, a fault in the grid could have propagated to the PV system, leading to a shutdown. A malfunction in the inverter could have prevented it from converting DC power from the PV modules to AC power for grid injection.

**Fig. 27:**
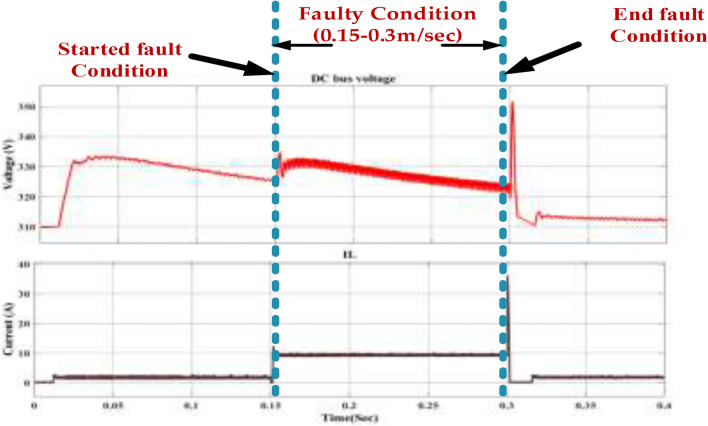
Wave forms of the DC bus voltage of the PMSM under healthy mode and faulty mode.

The fault occurs between 0.15 and 0.3 s. During this period, we observe the following changes in the system. The DC bus voltage experiences a significant dip during the fault. This indicates that the fault has disrupted the power flow to the DC bus, causing a voltage drop. The current also shows a sudden increase during the fault. This is likely due to the increased demand for the power source as the system tries to maintain the DC bus voltage. To prevent such faults and their consequences, several measures can be implemented. Regular inspection and maintenance of the DC bus components can help identify and rectify potential issues before they escalate into faults. Fault detection Isolation (FDI) systems can detect and isolate faults within the DC bus system, preventing them from affecting the entire system. Incorporating redundant components, such as capacitors or inductors, can improve system reliability and reduce downtime in case of faults. Using appropriate protection devices, such as fuses and circuit breakers, can help isolate faulty components and prevent the fault from spreading to other system parts, as represented in Fig. 27.

**Fig. 28:**
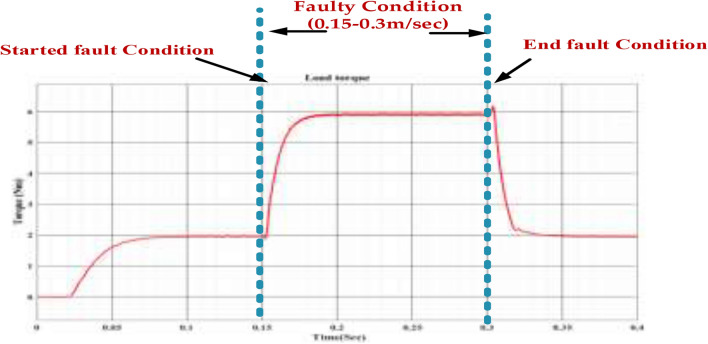
Wave forms of the load torque of the PMSM under healthy mode and faulty mode.

Figure 28 shows that the fault occurs between 0.15 and 0.3 s. During this period, we observe the following changes in the load torque. Before the fault, the load torque gradually increases, indicating that the system is accelerating. When the fault occurs, the load torque suddenly rises to a higher level. This could be due to several reasons. The fault might have caused an increase in the mechanical load on the system, requiring more torque to maintain the desired speed or operation. The fault might have destabilized the system, leading to torque and power demand oscillations. After the fault is cleared at 0.3 s, the load torque gradually decreases back to its pre-fault level. This indicates that the system is recovering from the fault and returning to its normal operating condition. Regular inspection and maintenance of the mechanical and electrical components can help identify and rectify potential issues before they escalate into faults. Incorporating redundant components, such as motors or drives, can improve system reliability and reduce downtime in case of faults. It is crucial to conduct a thorough investigation to determine the exact cause of the fault and take appropriate corrective actions.

## Real-time HIL results and discussions

**Fig. 29:**
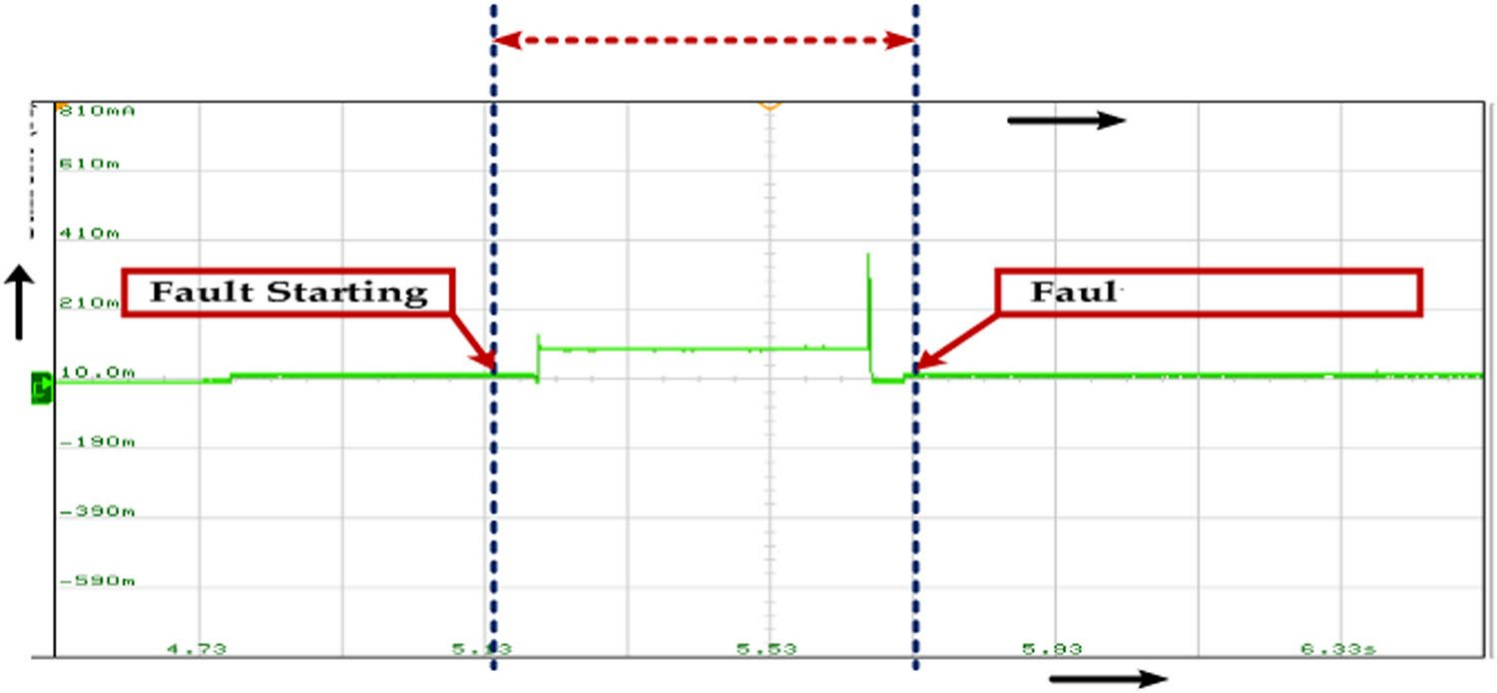
Wave forms of the stator current of the PMSM under healthy mode and faulty mode.

The sudden and significant increase in the stator current indicates the fault condition. The time duration of the fault is marked by “Fault Starting” and "Fault Completion." Fault Tolerance refers to the ability of a system to continue operating correctly even in the presence of a fault. In the context of stator current, it implies the system’s capacity to withstand the abnormal current surge without experiencing damage or malfunction. The given range of 0.15–0.3 s for fault tolerance suggests that the system should be able to handle the fault condition for at least 0.15 s and ideally up to 0.3 s, as presented in Fig. 29.

**Fig. 30:**
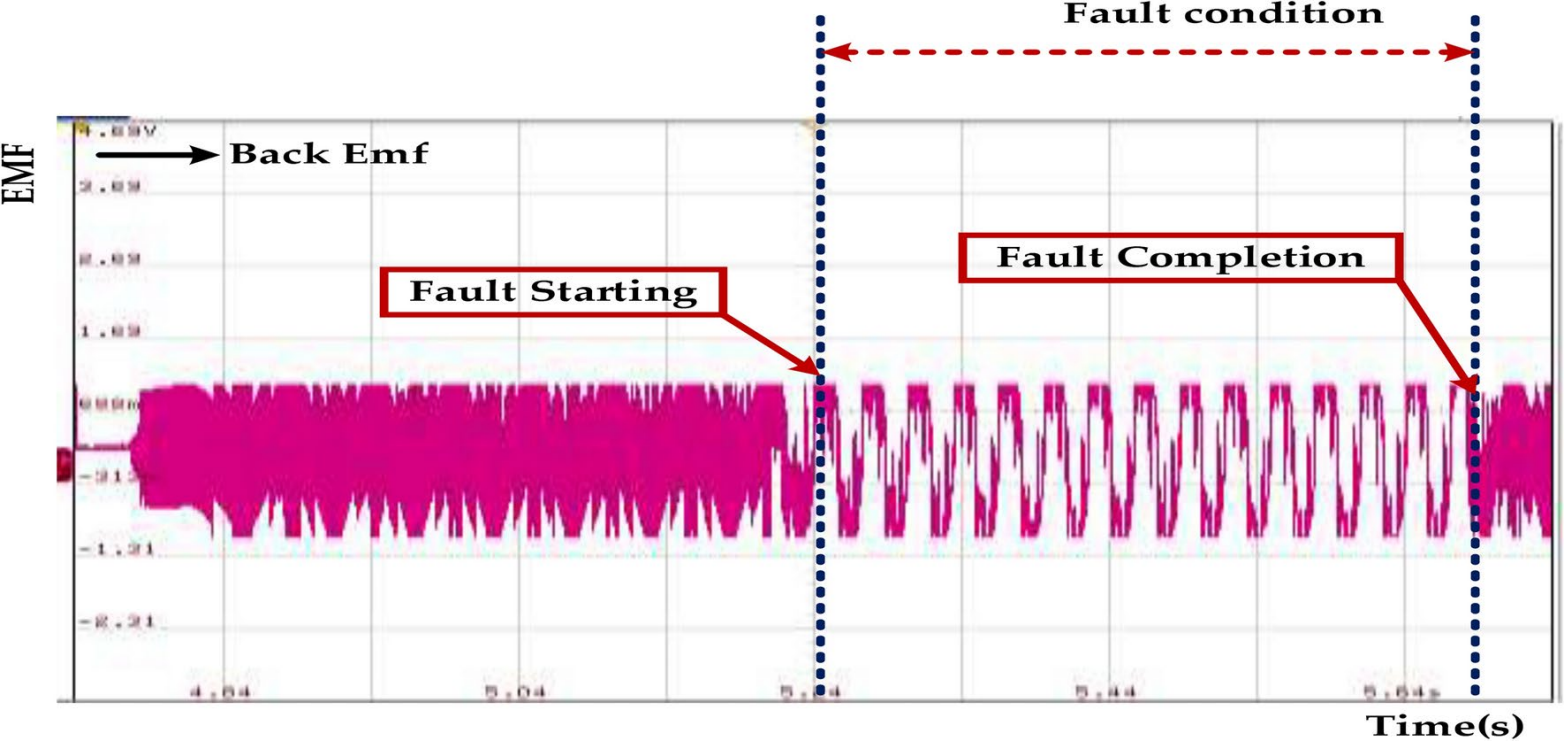
Open circuit inverter fault tolerance in back EMF of the PMSM under healthy mode and faulty mode.

Figure 30 represents Fault Tolerance, which refers to a system’s ability to continue operating correctly even in the presence of a fault. Back EMF (Electromotive Force) implies the system’s capacity to withstand the abnormal voltage fluctuations caused by the fault without experiencing damage or malfunction. Reliability ensures uninterrupted operation and minimizes downtime. Safety is protecting equipment and personnel from potential damage and hazards. Efficiency Reduces maintenance costs and extends the lifespan of the system. The given fault tolerance range of 0.15–0.3 s suggests that the system should be able to handle the fault condition for at least 0.15 s and ideally up to 0.3 s. The fault tolerance of 0.15–0.3 s indicates that the system is designed to handle short-duration faults effectively. However, it’s important to consider the specific fault type, system design, and protection measures to ensure optimal performance and reliability.

**Fig. 31:**
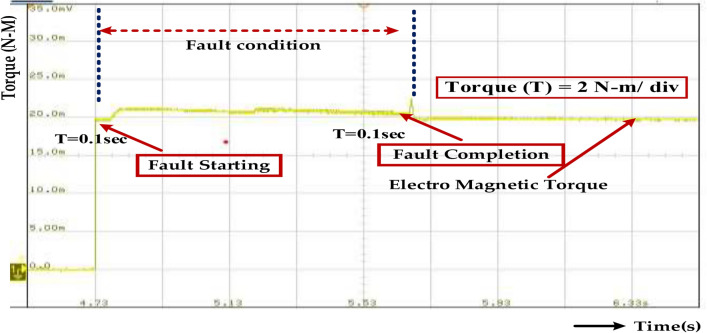
Wave forms of the electromagnetic torque of the PMSM under healthy mode and faulty mode.

Figure 31 represents the Fault Tolerance, which refers to a system’s ability to continue operating correctly even in the presence of a fault. In the context of torque, it implies the system’s capacity to withstand the abnormal torque fluctuations caused by the fault without experiencing damage or malfunction. The given range of 0.15–0.3 s for fault tolerance suggests that the system should be able to handle the fault condition for at least 0.15 s and ideally up to 0.3 s. The fault tolerance of 0.15–0.3 s indicates that the system is designed to handle short-duration faults effectively. However, it’s important to consider the specific fault type, system design, and protection measures to ensure optimal performance and reliability.

**Fig. 32:**
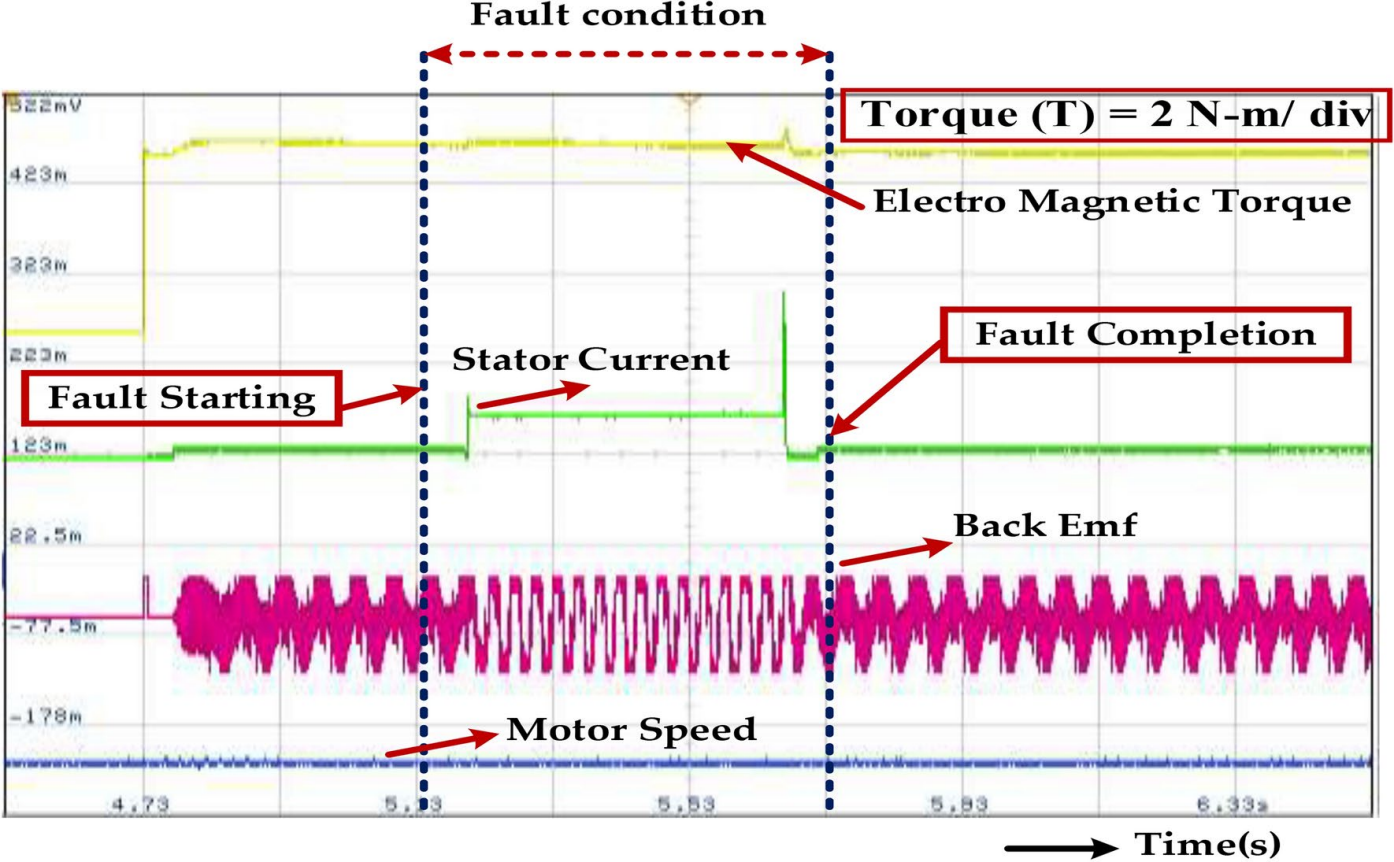
Wave forms of the stator current motor speed, back EMF, electromagnetic torque of the PMSM under healthy mode and faulty mode.

As mentioned earlier, the system’s fault tolerance refers to its ability to withstand the fault condition for a certain duration without experiencing catastrophic failure. The range of 0.15–0.3 s suggests that the system is designed to handle short-duration faults effectively. This illustrates a fault condition within the motor, characterized by abnormal changes in stator current, torque, Back EMF, and potentially motor speed. The fault duration is estimated to be between 0.15 and 0.3 s. This range indicates that the system’s fault tolerance suggests its ability to handle short-duration faults without major consequences. However, it is crucial to identify and address the root cause of the fault to prevent recurrence and ensure the system’s long-term reliability, which is present in Fig. 32.

**Fig. 33:**
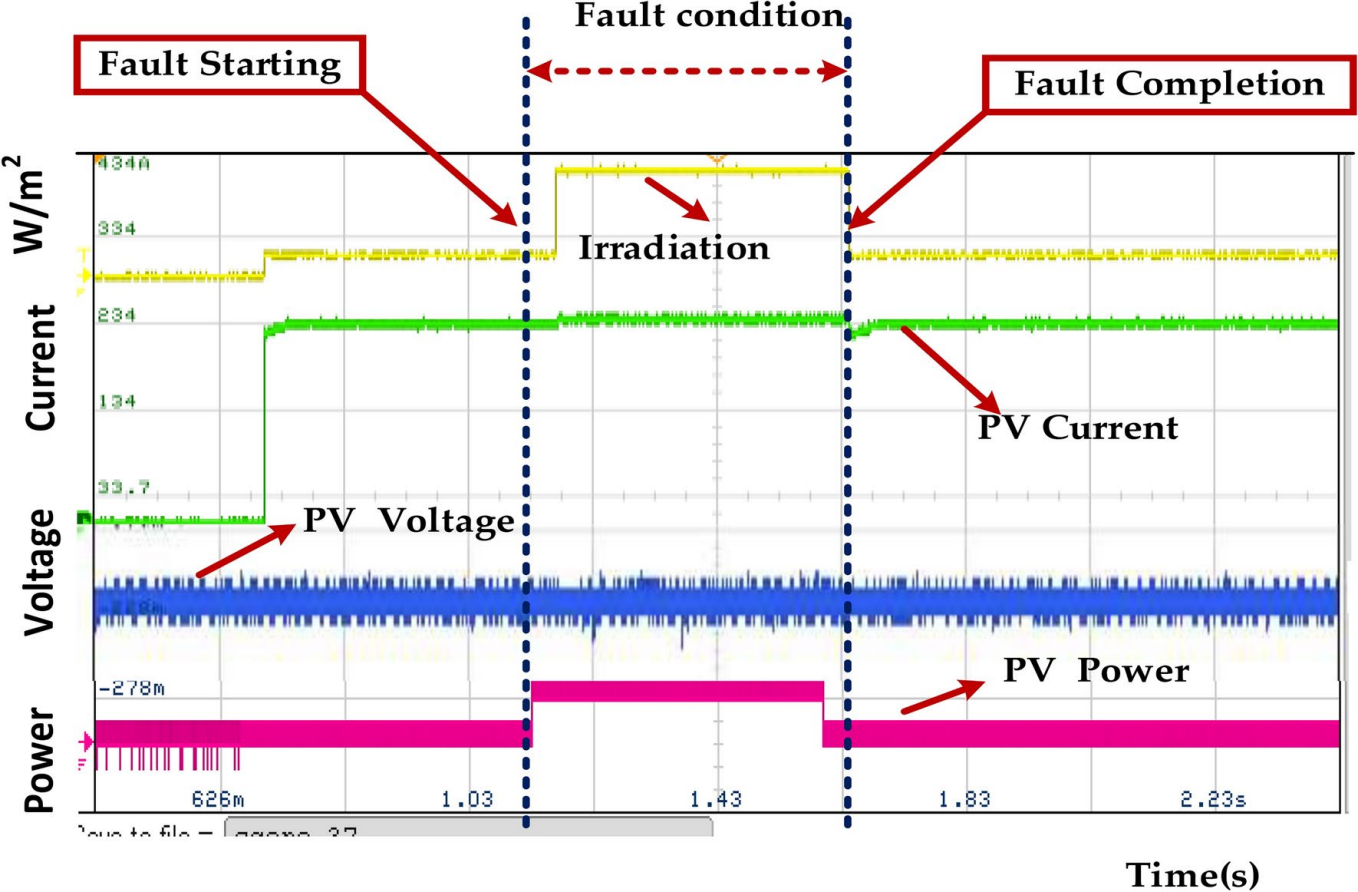
Wave forms of the solar PV voltage and current of the PMSM under healthy mode and faulty mode.

**Fig. 34:**
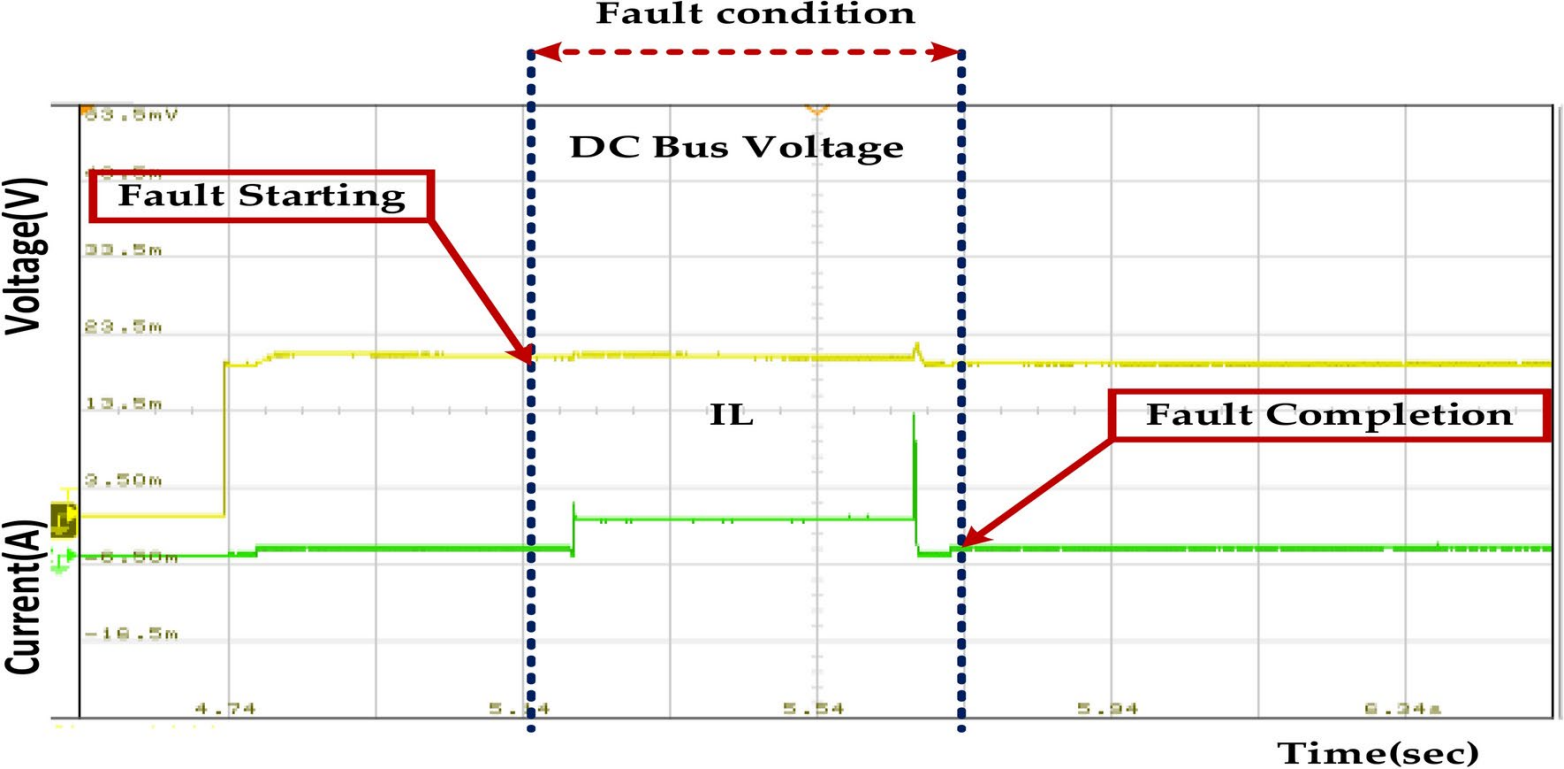
Wave forms of the DC bus voltage of the PMSM under healthy mode and faulty mode.

Figure 33 represents the sudden and significant changes in the following parameters, indicating the fault condition. Irradiation is a sudden decrease in irradiation is observed, which could be due to cloud cover or other environmental factors. PV Voltage: A corresponding reduction in PV voltage is seen, as lower irradiation results in reduced voltage output from the solar panels. The PV current also decreases due to the reduced irradiation and voltage. PV Power: The overall PV power output drops significantly due to the combined effect of lower voltage and current. The fault duration is estimated to be approximately 0.15–0.3 s, as indicated by the time markings on the graph. Figure 34 represents the DC Bus Voltage.

**Fig. 35:**
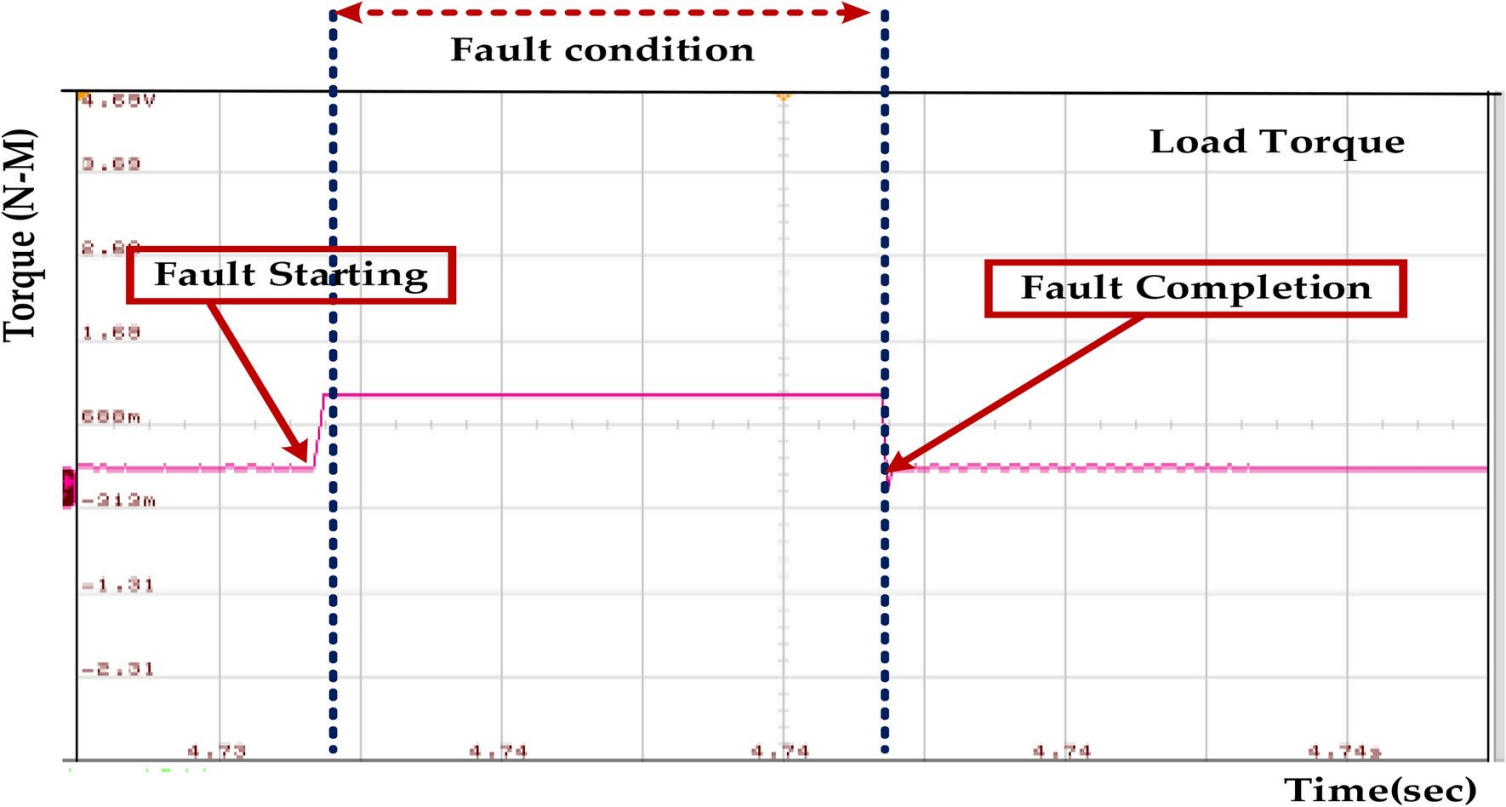
Wave forms of the stator of the PMSM under healthy mode and faulty mode.

Figure 35 represents sudden and significant changes in the load torque, indicating the fault condition. The torque initially remains constant, then experiences a rapid drop, then a recovery to a new steady-state value. The fault duration is estimated to be approximately 0.15–0.3 s, as indicated by the time markings on the above diagram. This illustrates a fault condition within the system, which is characterized by a sudden change in load torque. The fault duration is estimated to be between 0.15 and 0.3 s. This range indicates that the system’s fault tolerance suggests its ability to handle short-duration faults without major consequences. However, it is crucial to identify and address the root cause of the fault to prevent recurrence and ensure the system’s long-term reliability.

*OP5700 Real-Time HIL Simulator*: This device is likely responsible for generating the fault tolerance waveform and sending it to the oscilloscope. It acts as a hardware-in-the-loop (HIL) simulator, allowing for real-time testing and validation of control systems. This pin on the HIL simulator is connected to the oscilloscope, transmitting the fault tolerance waveform for visualization and analysis. The oscilloscope is used to capture and display the fault tolerance waveform. It can display both analog and digital signals, making it suitable for analyzing a wide range of waveforms. This software is likely running on the computer that controls the HIL simulator and generates the fault tolerance waveform. It provides a virtual environment for testing and simulating control systems.

**Fig. 36:**
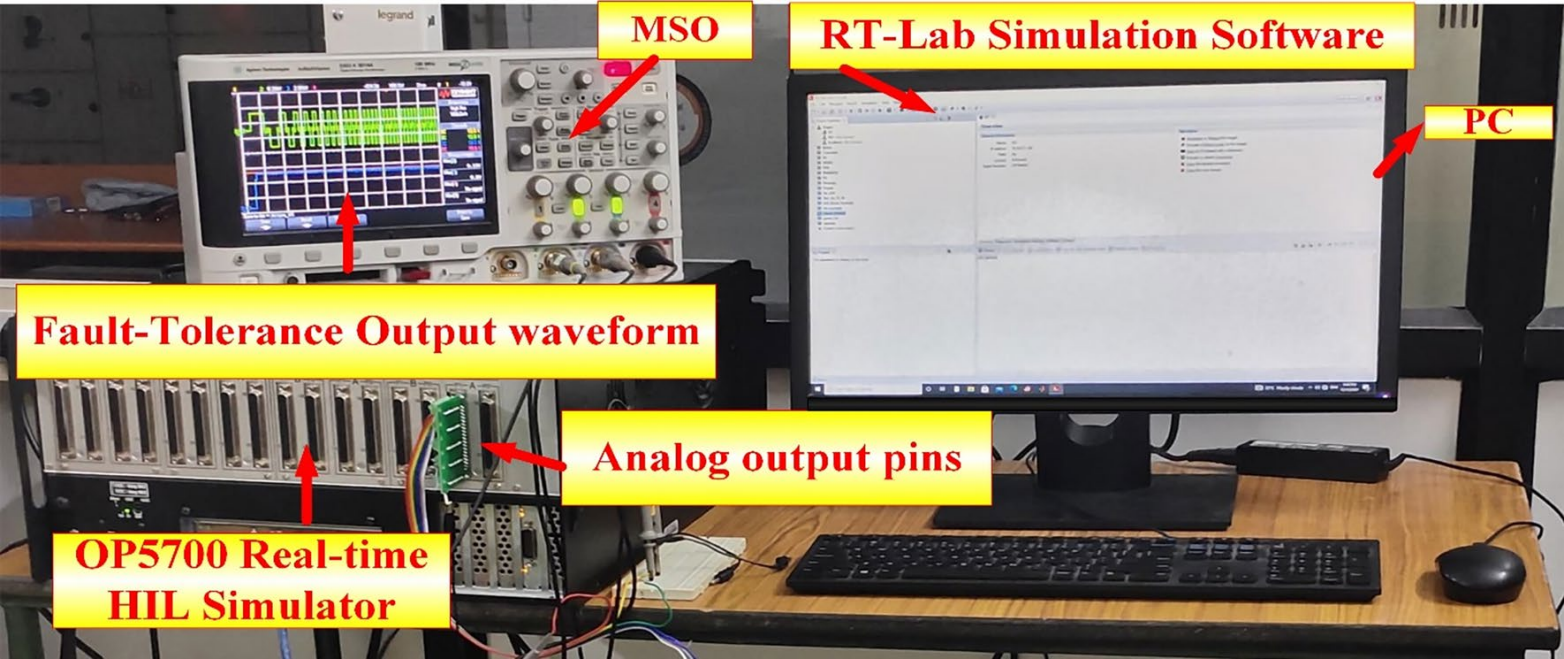
HIL simulator in real-time system setup.

The fault tolerance waveform displayed on the oscilloscope represents the output of the HIL simulator, which is designed to mimic the behavior of a real-world system under fault conditions. This waveform can assess the system’s response to various fault scenarios and evaluate its ability to maintain stability and performance. Figure 36 represents the system’s hardware in the loop (HIL) simulator. This setup is likely used for testing and validating the fault tolerance capabilities of a control system. Engineers can assess its robustness and identify potential weaknesses by generating fault scenarios and analyzing the system’s response. This information can be used to improve the system’s design and implementation. The HIL simulator provides a realistic environment for testing fault tolerance. The oscilloscope allows for visualization and analysis of the fault tolerance waveform. The RT-Lab simulation software controls the HIL simulator and generates the fault scenarios. This setup is valuable for evaluating the performance of control systems under fault conditions.

## Discussion

Simulations would accurately show the changes in stator current, back EMF, torque, and speed during the fault event. This would allow for developing and testing various fault detection algorithms (e.g., model-based, signal-based, AI-based). Simulations would enable detailed analysis of the fault’s impact on various motor parameters, including temperature rise, mechanical stress, and overall system performance. Simulations would allow for evaluating different fault tolerance strategies, such as control reconfiguration, redundancy techniques, and fault-tolerant control algorithms. This would help in selecting the most effective strategy for a given application. OPAL-RT would provide real-time monitoring of motor variables, allowing for immediate detection of faults. This would enable faster fault response and protection mechanisms. OPAL-RT would enable testing fault diagnosis and tolerance algorithms on real hardware, providing a more realistic evaluation of their performance under real-world conditions. OPAL-RT provides real-time testing, OPAL-RT provides real-time testing, which is crucial for evaluating the performance of fault diagnosis and tolerance techniques in real-time applications. While valuable for design and analysis, simulations may not accurately capture real-time dynamics.

## Conclusion

This paper investigates the application of time-based fault tolerance techniques in solar PV, DC–DC converter, battery, and motor systems. By employing switch-level, leg-level, module-level, and measurement-level techniques, the study focuses on early detection, isolation, and recovery from faults within a critical 0.15–0.3 s timeframe. Through detailed exploration and evaluation of the OPAL-RT platform, the research provides valuable insights into the behavior of these systems under fault conditions. The findings contribute to the advancement of fault-tolerant systems, offering practical guidance for the implementation and performance enhancement of time-based techniques in solar PV and motor applications. In the future, this paper will investigate the application of time-based fault tolerance techniques in solar PV, DC–DC converter, battery, and motor systems. By employing switch-level, leg-level, module-level, and measurement-level techniques, the study focuses on early detection, isolation, and recovery from faults within a critical 0.15–0.3 s timeframe. Through detailed exploration and evaluation of the OPAL-RT platform, the research provides valuable insights into the behavior of these systems under fault conditions. In addition, it also uses fewer TRIAC switches to bypass the gate signal from faulty switch to fault-tolerant one, reducing the losses in the power converter and thus increasing the overall efficiency of the power conversion system. The findings contribute to the advancement of fault-tolerant systems, offering practical guidance for the implementation and performance enhancement of time-based techniques in solar PV and motor applications. Leveraging machine learning algorithms to improve fault diagnosis accuracy and reduce response times. Deep Learning explores the potential of deep learning models to enhance fault diagnosis accuracy, especially for complex and multiple fault scenarios. Combine traditional signal processing techniques with machine learning to improve fault detection and isolation capabilities.

## Data Availability

The data used to support the findings of this study are included in the article.
